# Massive genome reduction predates the divergence of Symbiodiniaceae dinoflagellates

**DOI:** 10.1093/ismejo/wrae059

**Published:** 2024-04-24

**Authors:** Sarah Shah, Katherine E Dougan, Yibi Chen, Rosalyn Lo, Gemma Laird, Michael D A Fortuin, Subash K Rai, Valentine Murigneux, Anthony J Bellantuono, Mauricio Rodriguez-Lanetty, Debashish Bhattacharya, Cheong Xin Chan

**Affiliations:** Australian Centre for Ecogenomics, School of Chemistry and Molecular Biosciences, The University of Queensland, Brisbane, QLD 4072, Australia; Australian Centre for Ecogenomics, School of Chemistry and Molecular Biosciences, The University of Queensland, Brisbane, QLD 4072, Australia; Australian Centre for Ecogenomics, School of Chemistry and Molecular Biosciences, The University of Queensland, Brisbane, QLD 4072, Australia; Australian Centre for Ecogenomics, School of Chemistry and Molecular Biosciences, The University of Queensland, Brisbane, QLD 4072, Australia; Australian Centre for Ecogenomics, School of Chemistry and Molecular Biosciences, The University of Queensland, Brisbane, QLD 4072, Australia; Australian Centre for Ecogenomics, School of Chemistry and Molecular Biosciences, The University of Queensland, Brisbane, QLD 4072, Australia; Genome Innovation Hub, The University of Queensland, Brisbane, QLD 4072, Australia; Genome Innovation Hub, The University of Queensland, Brisbane, QLD 4072, Australia; Biomolecular Science Institute, Department of Biological Sciences, Florida International University, Miami, FL 33099, United States; Biomolecular Science Institute, Department of Biological Sciences, Florida International University, Miami, FL 33099, United States; Department of Biochemistry and Microbiology, Rutgers University, New Brunswick, NJ 08901, United States; Australian Centre for Ecogenomics, School of Chemistry and Molecular Biosciences, The University of Queensland, Brisbane, QLD 4072, Australia

**Keywords:** dinoflagellates, Symbiodiniaceae, genome evolution, free-living, symbiosis, coral symbionts

## Abstract

Dinoflagellates in the family Symbiodiniaceae are taxonomically diverse, predominantly symbiotic lineages that are well-known for their association with corals. The ancestor of these taxa is believed to have been free-living. The establishment of symbiosis (i.e. symbiogenesis) is hypothesized to have occurred multiple times during Symbiodiniaceae evolution, but its impact on genome evolution of these taxa is largely unknown. Among Symbiodiniaceae, the genus *Effrenium* is a free-living lineage that is phylogenetically positioned between two robustly supported groups of genera within which symbiotic taxa have emerged. The apparent lack of symbiogenesis in *Effrenium* suggests that the ancestral features of Symbiodiniaceae may have been retained in this lineage. Here, we present *de novo* assembled genomes (1.2–1.9 Gbp in size) and transcriptome data from three isolates of *Effrenium voratum* and conduct a comparative analysis that includes 16 Symbiodiniaceae taxa and the other dinoflagellates. Surprisingly, we find that genome reduction, which is often associated with a symbiotic lifestyle, predates the origin of Symbiodiniaceae. The free-living lifestyle distinguishes *Effrenium* from symbiotic Symbiodiniaceae vis-à-vis their longer introns, more-extensive mRNA editing, fewer (~30%) lineage-specific gene sets, and lower (~10%) level of pseudogenization. These results demonstrate how genome reduction and the adaptation to distinct lifestyles intersect to drive diversification and genome evolution of Symbiodiniaceae.

## Introduction

Dinoflagellates (Dinophyceae, Alveolata) are a diverse group of microbial eukaryotes that are ubiquitous in aquatic environments. Species in the family Symbiodiniaceae [1] comprise photosynthetic taxa that form symbioses with diverse marine organisms. Of particular importance to modern coral reefs, Symbiodiniaceae provide photosynthates via fixed carbon and essential nutrients to their cnidarian hosts. The Symbiodiniaceae ancestor is believed to have been free-living [1], with members of this group forming symbiotic associations with corals as early as 230 million years ago (MYA) [2]. Symbiogenesis, or the establishment of a symbiotic relationship between two or more taxa [3], can drastically influence lineage evolution, adaptation, and speciation as observed in obligate parasites and diverse symbiotic taxa [4]. This phenomenon is termed the resident genome syndrome and was previously hypothesized to explain the observed patterns of Symbiodiniaceae genome evolution [5].

Based on current divergence time estimates for Symbiodiniaceae, using large subunit rRNA via a clock model calibrated using coral fossil data [1], the split of genera *Symbiodinium* and *Philozoon* [1, 6] from other lineages occurred 166 ± 46 MYA; the later-branching symbiotic lineages diversified 109 ± 30 MYA [1]. If the emergence of symbiogenesis coincides with the earliest fossil evidence from 230 MYA [2], Symbiodiniaceae lineages would have arisen and diversified during major global geological events. These include the switch from aragonite to calcite seas (~190 MYA [7]), the breakup of Pangea (150–230 MYA [8]), the diversification or extinction of potential hosts such as rudists 66 MYA [9, 10], and the change in coral morphology from the Triassic (201–252 MYA) to the Cretaceous (66–145 MYA) [11, 12].

Symbiogenesis is expected to impact the genome evolution of symbionts within a broad spectrum of “facultativeness” that reflects the nature of the host association (i.e. with obligate free-living and obligate symbiont at opposing extremes). The symbiotic state underpins evolutionary processes such as genetic drift, expansion/contraction of mobile elements, pseudogenization, gene loss, and variation of mutation rates [5]. Previous studies that have investigated the effects of symbiogenesis on Symbiodiniaceae genomes have focused almost entirely on symbiotic genera, with the polar-dwelling, highly specialized *Polarella glacialis* [13], a sister of Symbiodiniaceae and within order Suessiales, comprising the sole, free-living outgroup.

Haploid genome sizes of Symbiodiniaceae taxa and *P. glacialis* are estimated to be <2 Gbp based on sequencing data [13–16], and <5 Gbp based on DNA staining and qPCR analysis of marker sequences [17, 18]. The taxonomically diverse dinoflagellates external to the Symbiodiniaceae are predominantly free-living and, in comparison, have massive genome sizes, e.g. 4.8 Gbp estimated from sequencing data for the bloom-forming *Prorocentrum cordatum* [19] and 200 Gbp based on DNA staining, for *Alexandrium tamarense* [20].

Symbiodiniaceae comprises at least 15 clades, with 11 named genera thus far [1, 6, 21, 22], supported by molecular, morphological, and ecological data [1]. Among Symbiodiniaceae taxa, *Effrenium* is a genus considered to be free-living [1]. The sole species, *E. voratum*, is globally distributed in temperate oceans with seasonal mean temperatures 15–26°C [1, 23]. Ubiquitous in the water column and on macroalgal surfaces [24], *E. voratum* can potentially form blooms [23]. Although occasionally found on the surface of marine organisms, *E. voratum* is not known to colonize any hosts intracellularly [23, 25]. Attempts to establish a symbiotic relationship with the anemone *Exaiptasia pallida* have been unsuccessful [26, 27]. Current understanding of Symbiodiniaceae evolutionary history suggests that *E. voratum* diverged 147 ± 40 MYA from the largely symbiotic genera of *Symbiodinium* and *Philozoon* during the early evolutionary history of Symbiodiniaceae [1]. Whereas genome data of other free-living species (e.g. *Symbiodinium natans*) are available, these taxa belong to genera that also include symbiotic species and thus might have experienced a symbiotic lifestyle at some point in their history. Based on these data, we posit that the genus *Effrenium* has remained unaffected by the influence of symbiogenesis and retains the free-living lifestyle, and therefore the ancestral genome features of Symbiodiniaceae. These genomic features provide a critical reference for understanding how multiple symbiogenesis events have contributed to the evolution of Symbiodiniaceae lineages to become successful symbionts in a wide range of hosts.

Here, we present *de novo* assembled genome and transcriptome data for three isolates of *E. voratum*. Incorporating publicly available genome-scale data from 16 Symbiodiniaceae taxa plus four free-living taxa external to the Symbiodiniaceae in a comparative genomic analysis, we examine genomic features in *E. voratum*. These include mobile elements, gene structures, gene-families, and pseudogenization to gain insights into ancestral features of Symbiodiniaceae.

## Materials and Methods

### Extraction of genomic DNA and total RNA

Cell cultures of *E. voratum* RCC1521, rt-383, and CCMP421 were provided by the LaJeunesse Lab (Pennsylvania State University). They were maintained using Daigo’s IMK medium (25°C, 14:10 h light–dark cycles).

For RCC1521 and rt-383, cells were pelleted by centrifugation (300 *g*, 5 min, room temperature [RT]), and resuspended in 100–500 μL pre-warmed (60°C) lysis buffer (100 mM Tris–HCl, 20 mM EDTA, 4% CTAB (w/v), 1.4 M NaCl, 1% PVP (w/v), 2% β-mercaptoethanol). This mixture was ground using a pre-chilled mortar and pestle, and high molecular-weight genomic DNA (gDNA) was extracted (https://dx.doi.org/10.17504/protocols.io.b5qyq5xw). For CCMP421, cells were pelleted and snap frozen in liquid nitrogen and ground (425–600 μm glass beads). DNA was extracted using the 2 × CTAB method [13]. The DNA was precipitated using chilled isopropanol, washed using chilled 70% ethanol, and stored in Tris–HCl (10 mM, pH 8) at −20°C until sequencing (Supplementary Methods, Supplementary Table 1).

To extract total RNA from RCC1521 for Iso-Seq sequencing, cell pellets were lysed (5× freeze–thaw cycles, 425–600 μm glass beads), before QIAGEN RNeasy Plant Mini Kit was used. To increase transcriptome diversity, we extracted more RNA using a second method (Supplementary Methods, Supplementary Table 1).

### Transcriptome assembly and processing

For RNA-Seq data, upon removal of adapters and unique molecular identifiers using bcl2fastq v2.20.0.422, the reads were trimmed and filtered using fastp v0.20.0 (*-A -L 35 -g -x --cut_front --cut_window_size 4 --cut_mean_quality 15*) and assembled using Trinity v2.9.1 [28] in “*de novo*” (*--SS_lib_type RF --trimmomatic*) and “genome-guided” modes; for “genome-guided”, RNA-Seq reads were mapped to the assembled genome using HISAT2 [29] before Trinity was run (*--SS_lib_type RF --genome_guided_bam --genome_guided_max_intron 70 000*). Raw Iso-Seq sequences underwent CCS generation and demultiplexing using the standalone modules CCS v4.2.0 and Lima v1.11.0, and high-quality transcripts were identified using the IsoSeq pipeline v3.3.0.

### *De novo* genome assembly

*De novo* genome assemblies were generated by combining Illumina, PacBio, and Nanopore data using MaSuRCA [30] v4.0.1 for RCC1521, and v3.4.2 for rt-383, with the built-in CABOG as the final assembler; the distinction between these two versions is the 6-fold decrease in run-time in v4.0.1 with negligible impact on the results. For CCMP421, the *de novo* genome assembly was generated from 10× Genomics linked-read sequencing data using Supernova v2.1.1 [31].

RCC1521 and rt-383 assemblies were scaffolded with L_RNA_scaffolder [32], using IsoSeq transcripts and *de novo* assembled transcripts from RNA-Seq (above). For CCMP421, linked-read distance information was used to refine the assembly with ARBitR v0.2 (*-m 27 k -s 10 k*) [33]. Due to the low quality of the publicly available transcriptome data of CCMP421 (i.e. MMETSP1110 [34] with only 54% mapped to the corresponding assembled genome; Supplementary Table 2), we used the *de novo* assembled transcripts from RCC1521 and rt-383 to scaffold the CCMP421 genome assembly via L_RNA_scaffolder.

We identified and removed putative sequences of bacterial or archaeal sources following a decision tree based on analysis using BlobTools v1.1 [35], yielding the final assembly for each isolate. Organellar genomic sequences were identified following a published method [13] (Supplementary Methods, Supplementary Tables 3–5). Completeness of each assembly was assessed using BUSCO v5.1.2 [36] against the alveolata_odb10 database (*genome* mode). Pairwise genome-sequence similarity was assessed using nucmer (*--mum*) implemented in MUMmer 4.0.0beta2 [37] at default setting. To predict protein-coding genes, we used a workflow customized for dinoflagellates [16], incorporating protein and transcriptome evidence and multiple predictors (Supplementary Methods and Supplementary Table 6).

### Inference of phylogenetic relationship

To infer species phylogenies using 18S rRNA genes and ITS2 markers, we identified these sequences from the *E. voratum* genome assemblies using BLASTn. Reference 18S rRNA genes (https://doi.org/10.5061/dryad.1717129 [1]; 79 sequences) and ITS2 sequences (https://symportal.org/ [38]; 8409 “published post-MED sequences”, 10 September 2021) were used for these analyses. For each marker sequence set, a multiple sequence alignment was generated using MAFFT v7.471 [39] (*mafft-linsi*) and trimmed using trimAl v1.4.rev15 [40] *(-automated1*), from which a maximum-likelihood tree was inferred using IQ-TREE v2.1.3 [41] (*-nm 2000 -bb 2000 -m MFP*). Putative orthologous protein sets identified from the 33 dinoflagellate taxa (Supplementary Tables 7 and 8) [19, 34, 42, 43] were used to infer a species tree, whereas whole-genome sequences were used in an alignment-free approach [44] to infer phylogenetic relationships from distinct genomic regions (Supplementary Methods, Supplementary Fig. 1, see online supplementary material for a colour version of this figure).

### Analysis of gene evolution of Suessiales taxa

We grouped 21 Suessiales protein sequence datasets into three groups: Ev, S1, and S2, with Po (*P. glacialis*) as outgroup (Supplementary Table 7). The total 811 661 protein sequences were clustered into homologous sets using OrthoFinder v2.5.4 [45]. Because these protein sets were generated solely based on sequence similarity, the lack of structural information in such an inference of homology may fail to recognize remote (i.e. highly divergent) homologs. However, these protein sets represent a proxy for studying the evolution of gene families, particularly in identifying putative gene gain or loss [45], and lineage-specific protein sets (Supplementary Methods).

### Identification of pseudogenes

Following an earlier study [15], pseudogenes were identified based on tBLASTn search using predicted protein sequences as query against the corresponding genome sequences for which gene model sequences were explicitly masked and excluded. Matched regions (≥75% identity) were considered fragments of pseudogenes; those at <1 Kb apart and in the same orientation were considered collectively as a pseudogene.

We focused on 752 954 protein sequences from 19 Suessiales taxa, specifically excluding *S. natans* and *Symbiodinium pilosum* from S1 to avoid signatures of free-living lifestyle in these taxa interfering with potential signatures of symbiogenesis. The protein sequences were clustered into homologous sets using OrthoFinder v2.5.4 [45]. We define the extent of pseudogenization, Ψ, as the ratio of the number of putative pseudogenes to the number of putative functional genes in a homologous set. We determined this value independently for Ev (Ψ_Ev_) against that for S1 (Ψ_S1_), S2 (Ψ_S2_), and the combined S1 and S2 (Ψ_S1 + S2_); a protein set with Ψ_S1_ > Ψ_Ev_ indicates a greater extent of pseudogenization in S1 than in Ev.

## Results

### Genomes of *E. voratum* isolates

We generated *de novo* genome assemblies for three isolates of *E. voratum* (assembly sizes 1.1–1.3 Gbp; Supplementary Methods, Supplementary Table 1), with estimated haploid genome sizes of 1.2–1.9 Gbp (Supplementary Table 9, Supplementary Fig. 2, see online supplementary material for a colour version of this figure), completeness (BUSCO recovery 67.2–77.2%), and number of predicted genes (32102–39 878) (Table 1, Supplementary Tables 7 and 10). The CCMP421 genome assembly, derived from 10× Genomics linked reads, is the most fragmented compared with RCC1521 and rt-383, which were derived from both short and long reads. BUSCO recovery of these assemblies is comparable to other published dinoflagellate genomes (55–70%; Supplementary Table 7).

**Table 1:** Genome assemblies and gene predictions of *E. voratum* RCC1521, rt-383, and CCMP421.

Isolate	RCC1521	rt-383 (=CCMP3420)	CCMP421
Location of isolation	Mediterranean Sea off Blanes, Spain [23]	Eastern North Pacific off Santa Barbara, USA [73]	Cooks Strait, New Zealand [74]
Genome sequencing technologies	Illumina, PacBio, Nanopore	Illumina, PacBio, Nanopore	10X Linked-reads
Genome assembly size (Gb)	1.2	1.3	1.1
Estimated genome size (Gb)	1.4	1.2	1.9
GC-content of genome assembly (%)	50.8	50.6	50.9
Total read coverage	446×	212×	153×
Number of genome scaffolds	3881	11 607	38 022
N50 of genome assembly (Kb)	720	252	304
Number of predicted genes	32 108	39 878	32 615
% BUSCO recovery (genome) alveolata_odb10; eukaryota_odb10	59.1; 29.4	62.0; 28.2	55.0; 20.0
% BUSCO recovery (protein) alveolata_odb10; eukaryota_odb10	76.1; 52.5	77.2; 54.1	67.2; 45.5

Genome sequences of the three isolates share high similarity (Supplementary Table 11) and exhibit conserved repetitive elements. For an in-depth pairwise genome-sequence comparison, including potential technical issues related to contiguity of these genome assemblies, see [46]. Repetitive regions containing protein-coding genes were highly conserved in *E. voratum* relative those of other Suessiales (the Order containing Symbiodiniaceae and the earlier branching sister *P. glacialis*). We identified 98 344 core *k*-mers (*k* = 23, all possible 23-base sequences; Supplementary Methods) that are common in genomes of all Suessiales taxa; 95% of core 23-mers were in repetitive regions of *E. voratum* (Supplementary Fig. 3, see online supplementary material for a colour version of this figure).

### Genome-size reduction pre-dated divergence of Symbiodiniaceae

**Figure 1:**
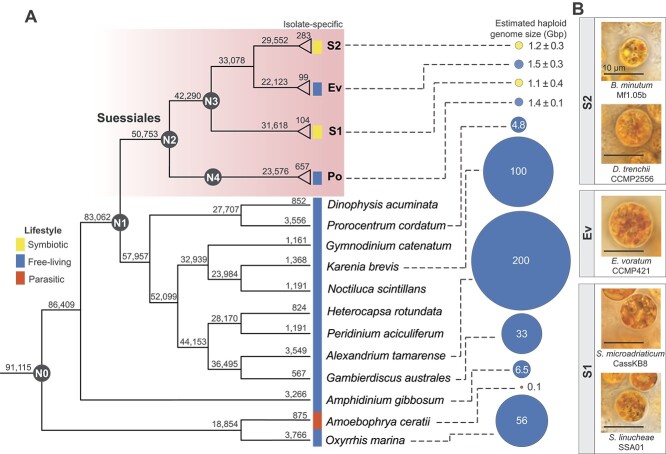
Species tree of dinoflagellates, showing estimated nuclear genome sizes; (A) the tree was inferred using 91 115 orthologous protein sets derived from 1 420 328 proteins from 33 dinoflagellate taxa, encompassing symbiotic, free-living, and parasitic lifestyles, and the number shown at each node denotes the number of homologous protein sets that are identified in any lineage descendant from that node, regardless of whether other lineages are also represented. The numbers at the terminal branches indicate protein sets that are recovered only in the specific lineages of the tip labels; Suessiales is shown as four dataset groups, i.e. the free-living Po external to the three Symbiodiniaceae clades of S1 (the early-branching, largely symbiotic *Symbiodinium*), Ev (the free-living *E. voratum*), and S2 (the later-branching symbiotic taxa). For S1, S2, Ev, and Po, the mean number of taxon-specific protein sets and the mean ± standard deviation of estimated genome sizes are shown. Ancestral nodes for all 33 taxa (N0), for Dinophyceae (core dinoflagellates; N1), for Suessiales (N2), for Symbiodiniaceae (N3), and for *Polarella glacialis* (N4) are indicated on the tree; and (B) the light micrographs of representative Symbiodiniaceae taxa in the dataset groups of S1, S2, and Ev are shown; scale bar = 10 μm.

For comparative genomic analysis, we obtained all available genomic data from 23 dinoflagellate taxa: 19 from Symbiodiniaceae [13, 15, 16, 47–51] (Order Suessiales), two sister taxa of *P. glacialis* [13] (Order Suessiales), and two distantly related free-living dinoflagellate taxa, *P. cordatum* [19] (Order Prorocentrales) and *Amphidinium gibbosum* [42] (Order Amphidiniales). Datasets from the 21 Suessiales taxa were grouped into: (i) the free-living outgroup (Po: *P. glacialis* strains CCMP1383 and CCMP2088) sister to Symbiodiniaceae, and among Symbiodiniaceae, (ii) the earlier-branching, largely symbiotic *Symbiodinium* (S1: *Symbiodinium linucheae*, *S. natans*, *Symbiodinium necroappetens*, *S. pilosum*, *Symbiodinium tridacnidorum* strains CCMP2592 and Sh18, and *Symbiodinium microadriaticum* strains 04-503SCI.03, Cass KB8, and CCMP2467), (iii) the three free-living *E. voratum* isolates (Ev), and (iv) the later-branching symbiotic lineages (S2: *Breviolum minutum*, *Cladocopium proliferum*, *Cladocopium* sp. C15, *Cladocopium* sp. C92, *Durusdinium trenchii* strains CCMP2556 and SCF082, and *Fugacium kawagutii*) (Supplementary Table 7). The phylogenetic positions of these groups relative to other dinoflagellates are shown in Fig. 1A, along with light micrographs of representative species in S1, S2, and Ev (Fig. 1B). Cell size of *E. voratum* (12.2–13.3 μm [23]) is generally larger than S1 (e.g. *S. microadriaticum* CassKB8; 8.0–11.0 μm [17]) or S2 cells (e.g. *D. trenchii* CCMP2556; 7.5–10.0 μm [17]).

The most striking feature of genome evolution among Suessiales is the marked reduction in genome size that occurred in the common ancestor of this lineage. Whereas free-living dinoflagellates external to the Suessiales have wide-ranging genome-sizes ca. 5–200 Gbp, except the parasitic *Amoebophrya ceratii* that has a highly reduced genome (0.1 Gbp), all Suessiales genomes fall within a much narrower size range from 0.7 to 2.0 Gbp, estimated using sequencing data (Fig. 1A). In an assessment of 1 603 073 protein sequences from 33 dinoflagellate taxa, we identified 50 753 homologous sets that contain one or more Suessiales taxa (node N2, Fig. 1A), compared with 83 062 sets that contain one or more Dinophyceae taxa (core dinoflagellates) [52] including Suessiales (node N1, Fig. 1A). This observation suggests streamlining of gene inventory and function in the evolution of Suessiales, particularly for Symbiodiniaceae, which may have driven symbiotic associations with cnidarians that offered nutrient-rich and protected habitats within the animal tissues. The facultative lifestyle was likely retained in most Symbiodiniaceae because it offers the benefit of sexual reproduction during the free-living stage [53]. The most substantial recovery from genome streamlining is through whole genome duplication, which has occurred in the *Durusdinium* lineage [48].

### Genomes of *E. voratum* have higher GC and longer introns than those of symbiotic lineages

**Figure 2:**
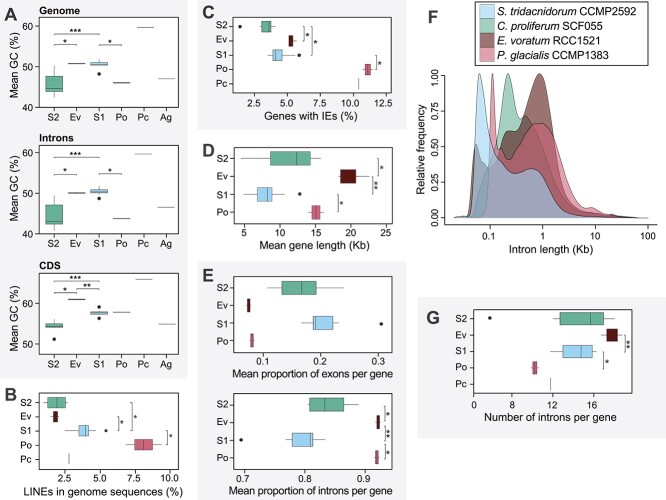
Genome features of *E. voratum* and other dinoflagellates; features of representative genomes in the four Suessiales groups of S2, Ev, S1, and Po, plus *P. cordatum* (Pc), in the order from the most-recent to most-ancient divergence, shown for (A) mean GC content in whole-genome, intronic, and CDS regions (*A. gibbosum* (Ag) was added for dinoflagellate-wide comparison), (B) percentage of mobile elements, and (C) percentage of genes containing IEs; gene features of Suessiales showing (D) mean gene length, (E) average proportions of exons and introns, (F) the relative frequency of introns by length (symbiotic lineages in boldface), and (G) number of introns per gene; in all bar charts, ^*^, ^*^^*^, ^*^^*^^*^ represent *P* < .05, <.01, and <.001, respectively, based on Wilcoxon rank sum test.

Compared with the genomes of symbiotic Symbiodiniaceae (i.e. S1 and S2), *E. voratum* genomes exhibit higher GC content, a comparable extent of mobile elements, and greater extent of introner elements (IEs). Overall, the GC content of coding regions varied among the Symbiodiniaceae lineages (Fig. 2A) and were lower in S2 (mean 54.2%; *P* < .05) relative to Ev (61.0%) and S1 (57.7%); in intronic regions, the mean GC is 44.6% (S2), 50.1% (Ev), and 50.4% (S1), whereas that of whole-genome sequences is 45.8% (S2), 50.7% (Ev), and 50.6% (S1). Variation of GC content in dinoflagellate genomes does not appear to correlate to lifestyle; among the free-living species external to Symbiodiniaceae, the genomes of *P. glacialis* and *A. gibbosum* have a mean GC content of 46.4%, similar to S2, whereas the genome of *P. cordatum* has the highest GC content described thus far for any dinoflagellate, at 59.7% [19]. Intracellular bacteria have a mutational bias toward low genomic GC content, e.g. ~20% [54], but intracellular eukaryotes display both low and high extreme GC content patterns, ranging as low as 24% in the malaria parasite *Plasmodium falciparum* [55] to 67% in the green alga *Chlorella variabilis* that is a symbiont in the ciliate *Paramecium* [56]. The lower GC content in S2 genomes than the S1 counterparts underscores the dynamic nature of genomic GC content evolution in intracellular eukaryotes. Based on the proportions of mobile elements in each group, more long interspersed nuclear elements (LINEs) were found in S1 (3.9%) than in Ev (1.8%) and S2 (1.9%) (Fig. 2B, Supplementary Fig. 4, see online supplementary material for a colour version of this figure, Supplementary Table 12). In addition, more (5%) Ev genes contain IEs, than do the S1 (4%) and S2 (3%) genes (Fig. 2C); mobility of these elements are thought to rely on transposases [57]. Our recovery of transposase sequences from most Symbiodiniaceae genomes (Supplementary Table 13, Supplementary Note) suggests a capacity for these IEs to be mobile.

Compared with genes in other Symbiodiniaceae, *E. voratum* genes are longer and contain longer introns. Significantly (*P* < .05) longer genes were observed in Ev (mean 20 Kb) than S1 (8 Kb) and S2 (11 Kb) (Fig. 2D), primarily driven by longer intron sizes (introns make up, on average, 92% of a gene [Fig. 2E]; sizes peak at 1 Kb [Fig. 2F]) and higher intron density per gene (mean of 18 for Ev, 14 for S1, 14 for S2) (Fig. 2G).

### Symbiogenesis shaped the evolution of Symbiodiniaceae genes

**Figure 3:**
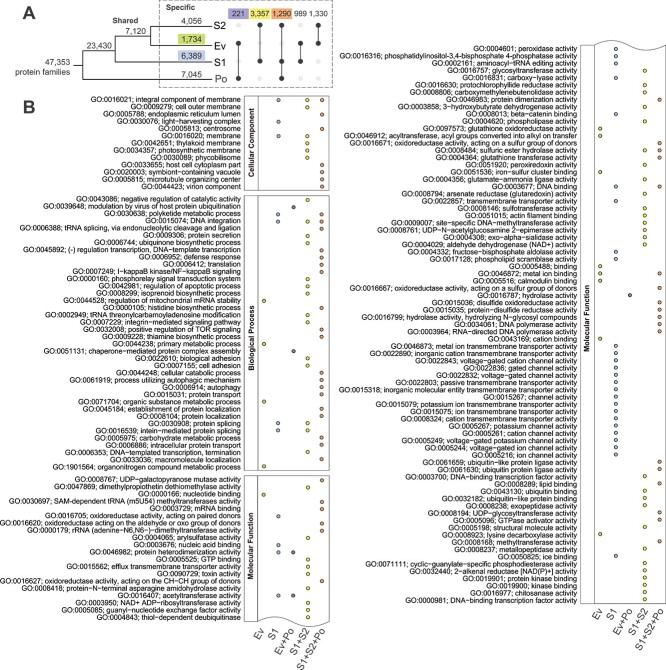
Evolution of Symbiodiniaceae genes; (A) the number of homologous protein sets is shown above each node and branch and represents those that are shared among or specific to S1, Ev, S2, and/or Po; number of protein sets that are exclusive to Ev, to S1, to Ev + Po, to S2 + S1, and to S2 + S1 + Po were highlighted, and (B) enriched gene ontology (GO) terms for genes in the five distinct groups relative to all GO terms in the corresponding taxa, arranged in decreasing order of significance from top to bottom within the categories: Cellular Component, Biological Process, and Molecular Function.

To examine the effect of a symbiotic lifestyle on the evolution of Symbiodiniaceae genes, we inferred 53 173 homologous sets from 811 611 protein sequences encoded by the genes in the 21 Suessiales genomes (see Materials and Methods). Dinoflagellate taxa external to Suessiales, for which genome data are limited, were excluded from this analysis. Most protein sets (47 353 of 53 173 [89%]) were shared among the four groups (S1 + S2 + Ev + Po). With respect to functions annotated in all homologous sets, these sets were enriched in functions such as cellular motility, biosynthetic processes for rRNA, antibiotics, and glycosides (Supplementary Table 14). There were more lineage-specific protein sets in S1 (6389) and S2 (4056) than in Ev (1734; Fig. 3A). The 3357 protein sets found only in S1 + S2 that split from each other over 40 million years of evolution may reflect convergent evolution due to the symbiotic lifestyle. These protein sets were enriched in diverse functions that include signalling, apoptosis, protein splicing, photosynthesis, cell adhesion, and various transferase activities (Fig. 3B). The protein sets specific to Ev were enriched in functions such as regulation of mitochondrial mRNA stability, metabolic processing of organic compounds, glutathione oxidoreductase, and the binding of calmodulin, metal ions, and nucleotides. This result highlights the importance of regulating energetic needs, metabolizing organic compounds, and the sequestration of metal ions in *E. voratum*, as a free-living Symbiodiniaceae. Incidentally, Po shared more protein sets with symbiotic lineages (1290; S1 + S2 + Po) than with Ev (221; Ev + Po) based on the datasets we analyzed here; the S1 + S2 + Po sets were enriched in functions such as autophagy and microtubule organization (Fig. 3B). Genetic duplication was found to drive intraspecific genomic divergence of Symbiodiniaceae [46], in which genes encoding functions related to photosynthesis were tandemly duplicated in genomes of the free-living *E. voratum* and *P. glacialis*. Although the number of lineage-specific homologous sets we recovered here is affected by the number and divergence of taxa represented in each group (Supplementary Methods and Supplementary Table 15), our observations reflect a higher divergence of genes within S1 and S2 (both comprising different species and genera) than in Ev (comprising a single species).

### Genomes of *E. voratum* exhibit less pseudogenization and greater RNA editing than those of symbiotic lineages

**Figure 4:**
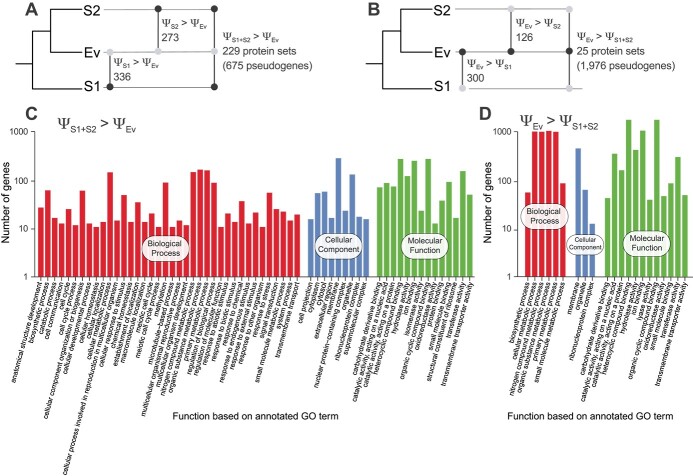
Pseudogenization in Symbiodiniaceae; the number of homologous sequence sets with significantly different levels of pseudogenization, Ψ, shown for (A) those with greater extent in symbiotic lineages (i.e. higher Ψ in S1, S2, or S1 + S2, relative to Ev), and (B) those with greater extent in the free-living Ev (i.e. higher Ψ in Ev relative to S1, S2, or S1 + S2); the black dots on the upset plots indicate taxa groups with higher Ψ than those with grey dots; the associated GO terms are shown for (C) those where Ψ_S1 + S2_ > Ψ_Ev_ and (D) those where Ψ_Ev_ > Ψ_S1 + S2_.

To test the hypothesis that facultative Symbiodiniaceae display higher levels of pseudogenization [5], we identified putative pseudogenes in Symbiodiniaceae genomes (Supplementary Table 16) based on shared sequence similarity of non-coding genomic regions to the predicted genes [15] (see Materials and Methods). In *E. voratum* RCC1521, rt-383, and CCMP421, we identified 42 462, 78 762, and 17 822 pseudogenes (Supplementary Table 16), compared with 32 108, 39 878, and 32 615 protein-coding genes (Table 1). For each taxon group, we assessed the level of pseudogenization as Ψ, the ratio of the number of pseudogenes to the number of genes in a homologous set (see Materials and Methods). We compared Ψ independently for Ev (Ψ_Ev_) against that for S1 (Ψ_S1_), S2 (Ψ_S2_), and the combined S1 and S2 (Ψ_S1 + S2_), then identified protein sets that exhibited significant difference (*P* < .05) of this value. More protein sets display Ψ_S1_ > Ψ_Ev_ (336) and Ψ_S2_ > Ψ_Ev_ (273; Fig. 4A), compared with Ψ_S1_ < Ψ_Ev_ (300) and Ψ_S2_ < Ψ_Ev_ (126; Fig. 4B). There was 9-fold more protein sets exhibiting Ψ_S1 + S2_ > Ψ_Ev_ (229; Fig. 4A) than vice versa (25; Fig. 4B). These pseudogenes are associated with a wide range of functions, including cell cycle processes and stimuli response (Fig. 4C and D). The protein sets that display significantly higher Ψ in the symbiotic lineages are mostly mutually exclusive from the 3357 sets that putatively experienced convergent evolution (only 16–23 sets are represented in Ψ_S1_, Ψ_S2_, Ψ_S1 + S2_). We found negligible technical biases in the clustering of homologous sequences that may affect our inference of pseudogenes (Supplementary Fig. 5, see online supplementary material for a colour version of this figure). These results suggest that in addition to potential convergent evolution in the symbiotic lineages, these Symbiodiniaceae have experienced a greater extent of pseudogenization than has the free-living Ev. Here, we excluded the free-living *S. natans* and *S. pilosum* from S1 to avoid signatures of their free-living lifestyle interfering with potential signatures of symbiogenesis in this group. We recovered fewer pseudogenes in these taxa (49 509 in *S. natans* and 16 607 in *S. pilosum*; Supplementary Table 16), when compared with the genomes of seven other symbiotic *Symbiodinium* taxa (mean 58 502). This result lends further support to the hypothesis of greater pseudogenization in genomes of symbiotic taxa when compared with those free-living, despite the variable quality of genome assemblies in our dataset (Supplementary Table 7).

We recovered greater extent of mRNA editing in *E. voratum* (45 009 sites implicating 28.5% of protein-coding genes) compared with the symbiotic taxa (e.g. 4227 sites implicating 7.6% of genes in *D. trenchii*; Supplementary Note, Supplementary Table 17, and Supplementary Fig. 6, see online supplementary material for a colour version of this figure). This observation is consistent with data from another free-living dinoflagellate [19], suggesting a more-pronounced role of mRNA editing in generating functional diversity in free-living versus symbiotic dinoflagellate taxa. However, the function and potential regulatory roles of these mRNA edited sites will need to be validated using targeted experiments [58]. Our alignment-free phylogenies (Supplementary Note, Supplementary Figs 7 and 8, see online supplementary material for a colour version of these figures) of the non-coding and repetitive genomic regions showed divergence of the branch containing Ev to be earlier than S1/S2 with robust node support, in contrast to the phylogeny inferred using the standard molecular markers of 18S rRNA (Supplementary Fig. 7A, see online supplementary material for a colour version of this figure), ITS2 (Supplementary Fig. 8A, see online supplementary material for a colour version of this figure), and multiple homologous protein sets (Fig. 1A). This result clearly indicates differential selective pressure acting on coding versus non-coding regions in Symbiodiniaceae genomes [44] driven by adaptation that involves incomplete lineage sorting, horizontal gene transfer, hybridization, and/or convergent GC-biased gene conversion [59, 60]. It may also reflect the retention of ancestral non-coding regions in the *E. voratum* genomes and/or the loss of some non-coding regions in symbiotic lineages due to genome streamlining. However, the direct impact of niche specialization of *E. voratum* on our observations remains to be investigated when more genome-scale data are available.

## Discussion

**Figure 5:**
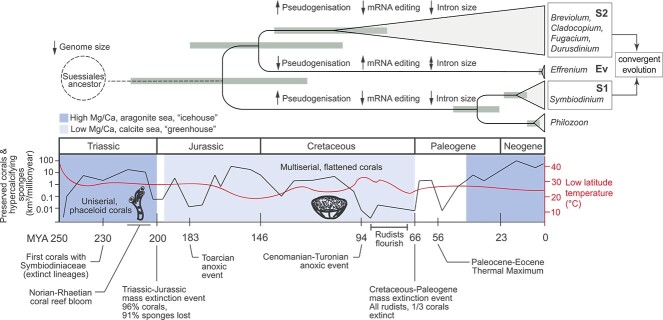
Timeline of Symbiodiniaceae genome evolution and coral evolution; the estimated divergence timeline of the family Symbiodiniaceae is shown at the top, indicating representative taxa of S1, Ev, and S2; grey bars represent 95% confidence intervals of divergence times, and key genome signatures for each group related to pseudogenization, mRNA editing, and intron size are shown along the branch; the dotted line represents the yet-unknown timeline of Suessiales divergence from the rest of dinoflagellates, and evolutionary timescale along the different eras highlighting key geological events relevant to coral evolution (adapted from Pandolfi and Kiessling [11]), aligning with Symbiodiniaceae divergence, is shown at the bottom; mass of preserved corals and sponges represented by black line (left *y*-axis), low latitude (30°S–30°N) ocean temperature in red smoothed line (right *y*-axis), ocean chemistry of Mg/Ca ratio showing aragonite (dark blue) vs calcite sea (light blue); data were sourced from earlier studies [1, 2, 10, 12, 75, 76].

The shared and distinct genomic features we observed between early- and later-branching symbiotic lineages of Symbiodiniaceae suggest an interplay between the geological eras during which they arose, and the corresponding coral morphology and ocean chemistry (Fig. 5). Ancestral Symbiodiniaceae inhabited stony corals presumably as early as 230 MYA in the late Triassic and may have driven the Norian-Rhaetian reef bloom [61]. These early Scleractinian corals (e.g. *Retiophyllia*) tended to be uniserial, i.e. possessing one corallite per branch and phaceloid with thick walls [62], and thus were less efficient at harvesting light [63]. The ability of the extant *Symbiodinium* to thrive under high or variable light [1] may be a trait inherited from their ancestor living in these ancient corals. Because these early Symbiodiniaceae adapted to different hosts, they likely underwent genome streamlining [5], experienced high pseudogenization and a reduction in mRNA editing and intron sizes (Fig. 5), as observed here and in other studies [15, 64]. Although these trends were also observed in the later-branching symbionts, *Symbiodinium* uniquely retains ancestral LINE repeats (Fig. 2C) which were lost in later-branching Symbiodiniaceae, including *E. voratum.*

*Effrenium voratum* is estimated to have diverged 147 ± 40 MYA in the late Jurassic [1], during the early diversification of Symbiodiniaceae. We did not find evidence for genome streamlining in *E. voratum*, but instead found genomic hallmarks associated with earlier-branching free-living lineages external to family Symbiodiniaceae, e.g. more IE-containing genes and larger intron size than in S1 and S2. Given that *Effrenium* is also the sole genus that is free-living within Symbiodiniaceae, we postulate that this lineage has not been impacted by symbiogenesis, in contrast to S1 or S2. Since the lineage diverged from S1, global events such as the breakup of Pangea and the Cretaceous-Paleogene mass extinction have occurred, along with changes in coral reef biomass (Fig. 5). Without additional evidence from the fossil record or ancient DNA analysis (e.g. the oldest evidence of Suessiales is *P. glacialis* from just 9000 years ago [65]), we cannot explain why *Effrenium* retained a free-living lifestyle. *Effrenium* and most other Symbiodiniaceae lineages (including free-living species from *Symbiodinium*) can form endolithic relationships with bacteria, e.g. as calcified biofilms [66], but why *Effrenium* apparently cannot form an endosymbiotic relationship with a host is unknown.

At the estimated time when S2 lineages diversified (109 ± 30 MYA) [1], shallow-water corals were multiserial, flatter, and more efficient at harvesting light [63]. This is coincident with a rise in ocean temperature (i.e. “greenhouse Earth,” when no continental glaciers existed) and the switch in ocean chemistry from an aragonite sea to a calcite sea, which would have made it difficult for corals to build their aragonite skeletons [11] (Fig. 5). In contrast, bivalve rudists that could build aragonite or calcite shells [67] radiated and flourished [10, 12]. These taxa likely harboured photosymbionts [9], presumably ancestral Symbiodiniaceae, given that extant Symbiodiniaceae (e.g. *S. tridacnidorum*) can inhabit modern bivalves [47]. Although the genomes of S2 have a lower GC content than those of S1, our results indicate that both S1 and S2 underwent genome streamlining, which may have led to convergent evolution of genes associated with functions relevant to forming a symbiotic association, such as cell signalling, apoptosis, and photosynthesis (Fig. 3). Our observation of longer introns in Ev than in S1/S2 could be explained by two possible evolutionary scenarios: (i) intron expansion in Ev or (ii) intron contraction in S1/S2. In the first scenario, TE-mediated insertions drove intron expansion and were biased toward the 5′-end of genes to prevent disruption of functional elements [68], yielding larger intron sizes in these regions. We did not observe this trend in Ev (Supplementary Fig. 9, see online supplementary material for a colour version of this figure). In the second scenario, which has been observed in endosymbiotic/parasitic organisms, the reduction of intron size and density occurs as a result of genome reduction and/or streamlining induced by spatial confinement in the host organism or cell [69]. Considering the evolutionary history of Symbiodiniaceae, intron contraction in S1/S2 taxa due to their symbiotic lifestyle is a more-plausible explanation for the data than intron expansion in Ev. Large introns observed in Po (Fig. 2F), albeit at a lower intron density per gene (Fig. 2G), lend further support to this notion. Therefore, we posit that symbiogenesis drove genome evolution in Symbiodiniaceae and elicited common features such as pseudogenization, lowered mRNA editing and intron contraction, but some features (e.g. LINE retention and GC content) were affected differently in earlier versus later-branching symbiotic lineages.

Based on the taxa we studied here, our results provide strong evidence for a phase of genome reduction that occurred in the Suessiales ancestor. This pattern is reminiscent of cyanobacterial lineages which have undergone gene loss, namely *Prochlorococcus* species when compared with their sister group, *Synechococcus* [70]. Both lineages inhabit oligotrophic, open oceans and do not exhibit drastic phenotypic differences, despite the significant changes that have occurred in genome content and organization. A more-extreme scenario is provided by the red algae (Rhodophyta), whose common ancestor underwent massive genome reduction, precipitating the loss of canonical eukaryotic features such as flagellum-based motility, phytochromes, and autophagy [71]. These gene losses predated the split of the two monophyletic lineages that comprise this phylum: the extremophilic Cyanidiophytina that specialized to life in hot spring environments and the species-rich mesophilic lineages (e.g. red seaweeds) that inhabit a variety of aquatic environments. Most red algae have therefore smaller genomes when compared with the green lineage and have adapted to diverse habitats through gene family evolution and horizontal gene transfer.

In an analogous fashion, it appears that the common ancestor of Suessiales underwent significant genome reduction. An alternate explanation for our data is that genome size reduction in the psychrophilic *P. glacialis* may have resulted from independent genome streamlining in this branch of evolution (Fig. 1) due to its highly specialized lifestyle and is unrelated to symbiosis or the ancestral state in Symbiodiniaceae. Under this scenario, two independent phases of genome reduction occurred, once in the Symbiodiniaceae ancestor (N3) and once in the *Polarella* lineage (N4; Fig. 1A). We favour (but cannot prove) the more parsimonious hypothesis of a single major genome reduction event (N2), followed by a more minor event that occurred in the *Polarella* branch (N4), with the latter driven by adaptation to a psychrophilic lifestyle (Fig. 1A). This type of scenario also played out in the Rhodophyta, in which there are two cases of genome reduction, a massive one in the red algal ancestor and a smaller one in the Cyanidiophyceae that reflects its transition to an extremophilic lifestyle [71]. Therefore, we hypothesize that the Symbiodiniaceae have smaller genome sizes than most free-living dinoflagellates, not because of the coral symbiosis, but due to more ancient selective constraints.

Our results are consistent with (but do not prove) the appealing idea that symbiosis offered an “escape” from reduced functional capacity due to genome reduction during the early stages of Symbiodiniaceae evolution; see [72] for a perspective. Regardless, the mixture of obligate free-living to facultative lifestyles among extant Symbiodiniaceae has resulted in divergent paths of genome evolution [53]. These results demonstrate the retention of ancestral Symbiodiniaceae genome features in *E. voratum* (in contrast to symbiotic lineages) despite multiple emergences of symbiogenesis over the past 200 million years. These observations support the notion that evolution favoured a free-living lifestyle for *E. voratum* (and by extension the genus *Effrenium*), likely due to local selective pressures. Therefore, *Effrenium* presents a useful free-living outgroup for studying the structural and functional genome features of symbiotic Symbiodiniaceae, and the implications of these features on ecology and evolution, including but not limited to host specificity and the facultativeness of symbiotic associations.

## Supplementary Material

Shah_SupplementaryFigures_1-9_ISMEJ_R3_wrae059

Shah_SupplementaryTables_1-17_ISMEJ_R2_wrae059

Shah_SupplementaryText_ISMEJ_R3_Final_wrae059

## Data Availability

All sequencing data generated from this study are available on NCBI GenBank via BioProject accession PRJEB61191. The assembled and annotated genomes for the three *E. voratum* isolates are available on GenBank (accessions GCA_963377175, GCA_963377275, and GCA_963377065). The assembled genomes, predicted gene models and proteins, identified organellar genome sequences, functional annotation of gene models, and the scripts associated with key analyses are available at https://doi.org/10.5281/zenodo.10894296. The scripts for complete genome annotation workflow of each *E. voratum* genome is available at https://doi.org/10.5281/zenodo.10896466 (RCC1521), https://doi.org/10.5281/zenodo.10896474 (rt-383), and https://doi.org/10.5281/zenodo.10896494 (CCMP421).
