# Supplementary material for: Massive genome reduction predates the divergence of Symbiodiniaceae dinoflagellates
Source: ISME J. 2024 Apr 24;18(1):wrae059. doi: 10.1093/ismejo/wrae059 (PMC11114475; doi:10.1093/ismejo/wrae059)
Supplement: Shah_SupplementaryFigures_1-9_ISMEJ_R3_wrae059 [file shah_supplementaryfigures_1-9_ismej_r3_wrae059.pdf]

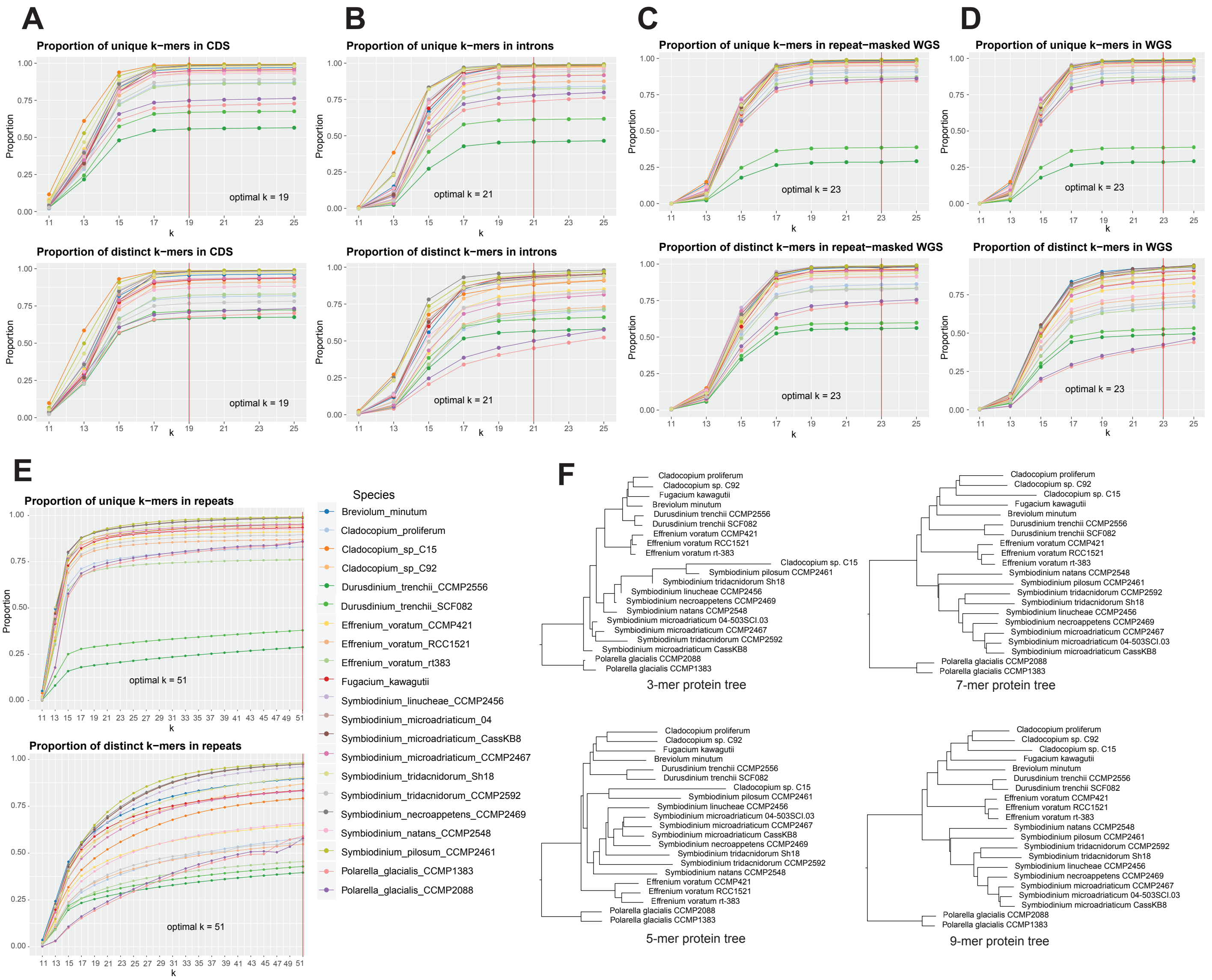

Supplementary Fig. 1. Proportions of unique and distinct *k*-mers extracted from Suessiales shown for (A) CDS, (B) introns, (C) repeat-masked whole genome sequences, (D) whole genome sequences, and (E) repetitive regions, with the corresponding optimal *k* marked with a red line, and (F) the four protein trees inferred based on distinct values of *k*.

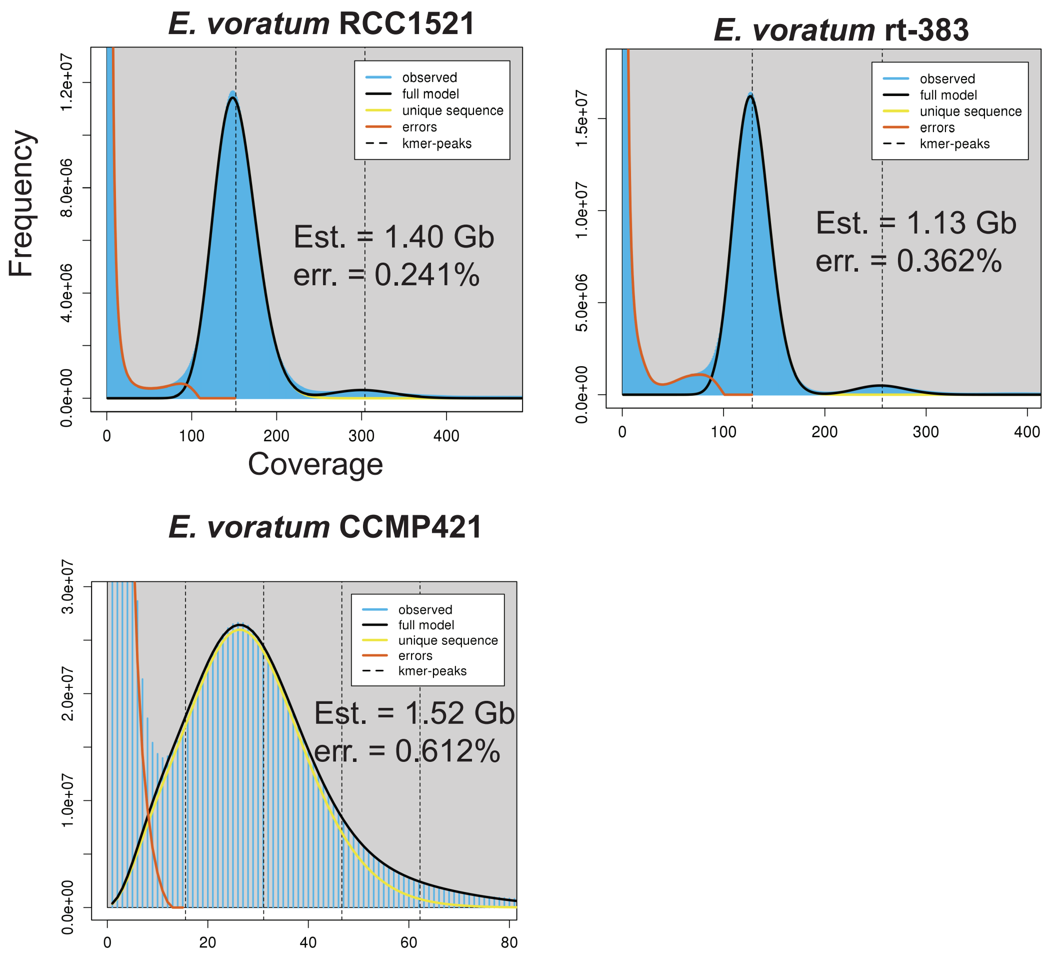

Supplementary Fig. 2. GenomeScope profiles of 21-mers from genomic sequence reads of *E. voratum* showing the fit to a haploid model and its error rate, and the estimated haploid genome size.

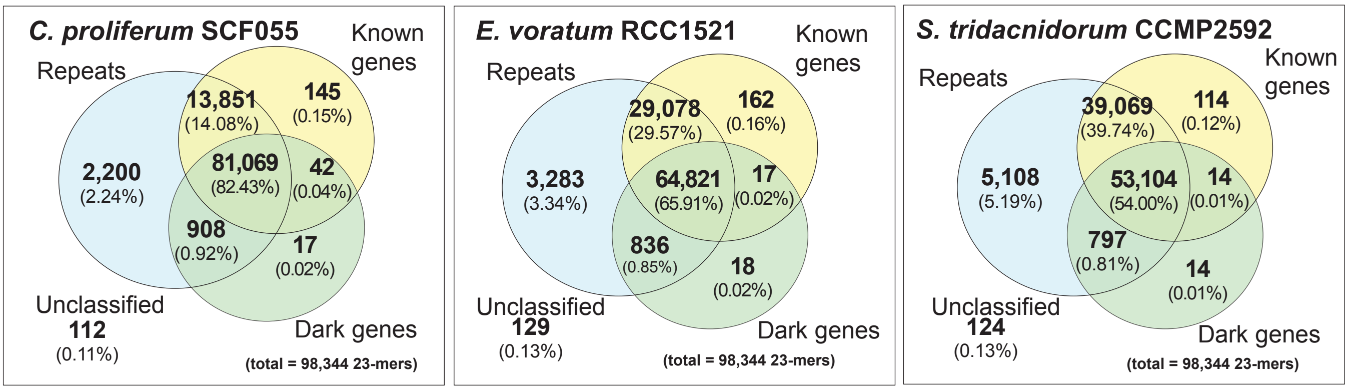

Supplementary Fig. 3. The proportion of core *k*-mers among the 21 Suessiales taxa that correspond to different genomic regions of known genes (yellow), genes of unannotated functions (green), and repeats (blue) in *C. proliferum*, *E. voratum*, and *S. tridacnidorum*.

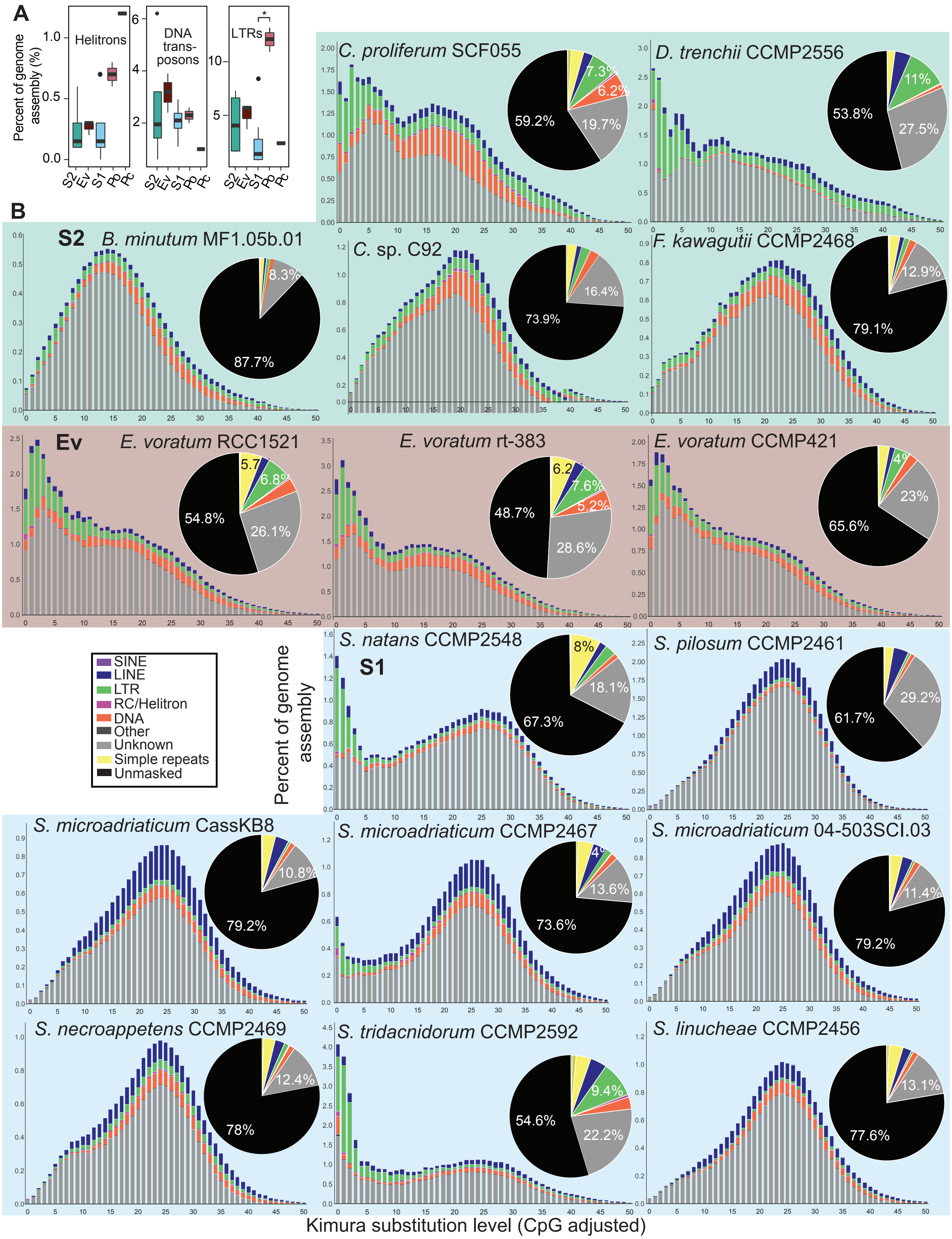

Supplementary Fig. 4. Mobile elements in dinoflagellates, showing (A) proportions of helitrons, DNA transposons, and LTRs in S2, Ev, S1, Po, and *Prorocentrum cordatum* (Pc), and (B) repeat landscapes in genomes of Symbiodiniaceae, for S1, Ev and S2, showing the percentage of repeat types in each genome assembly and their level of conservation (Kimura substitution level).

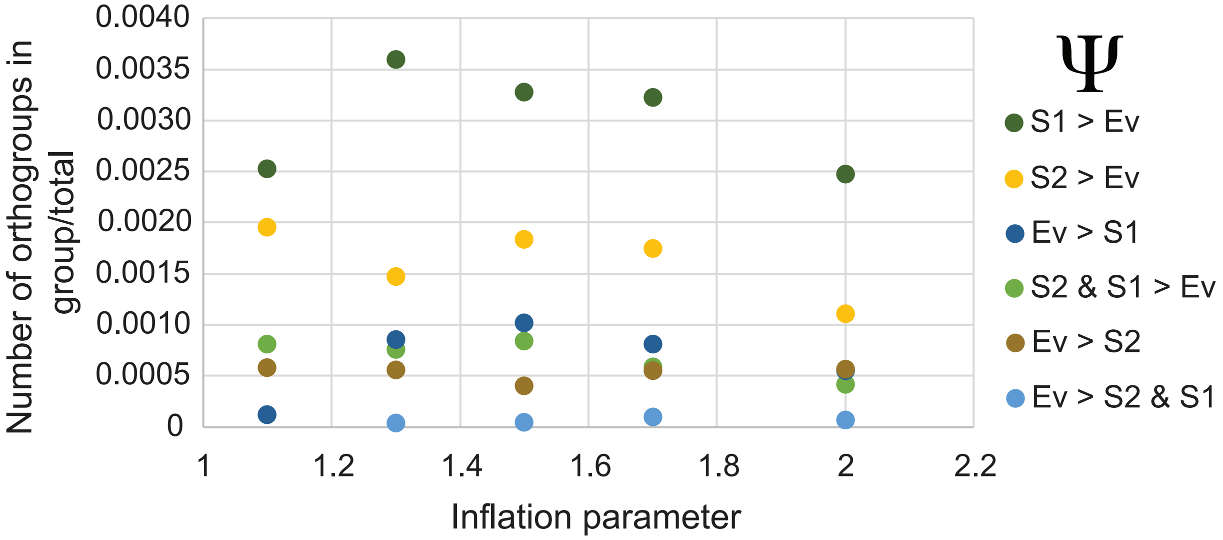

Supplementary Fig. 5. The proportion of homologous groups in the distinct groupings of pseudogenisation analysis identified at varied inflation parameters implemented in OrthoFinder.

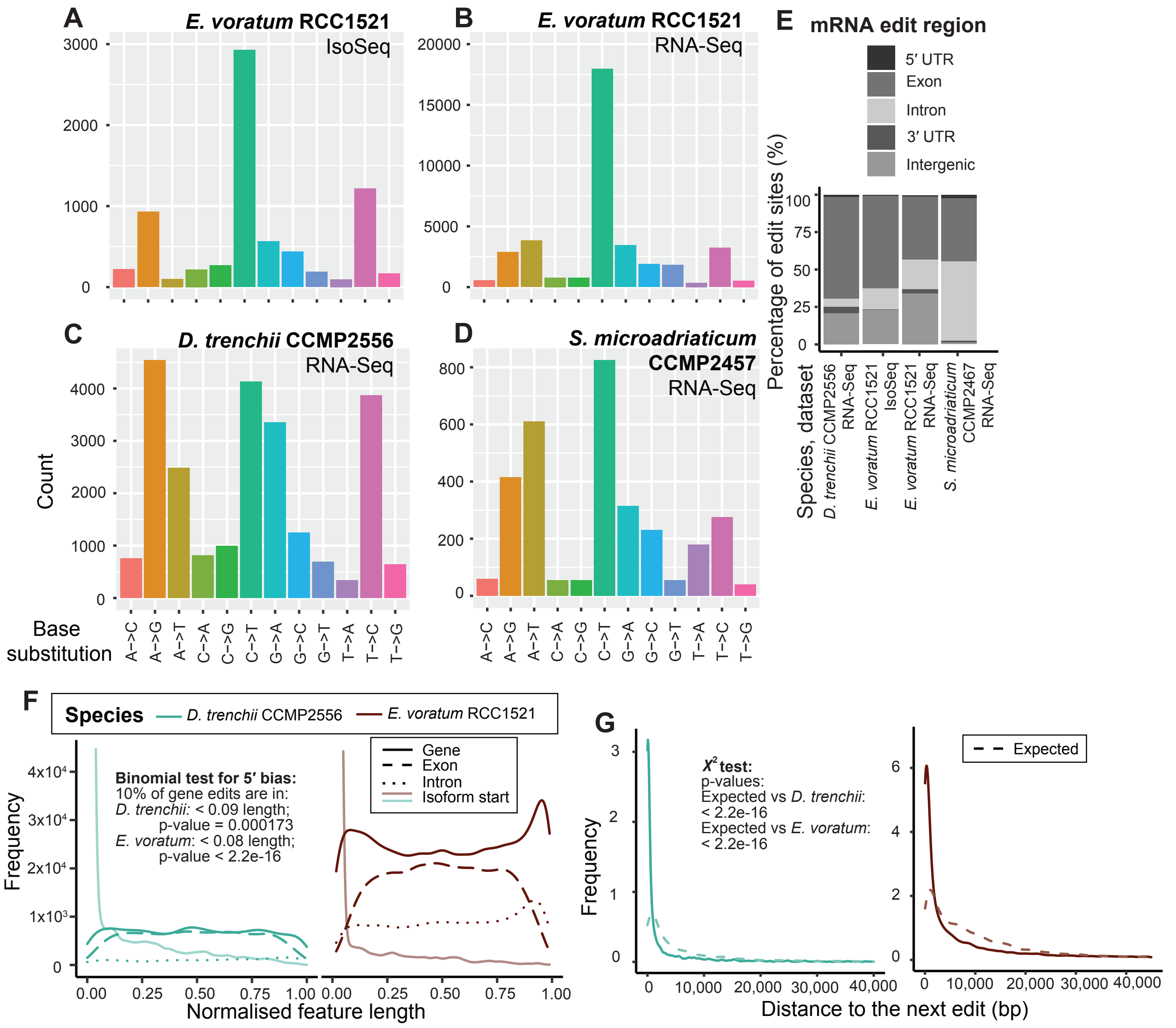

Supplementary Fig. 6. Editing of RNAs in dinoflagellates showing different edit types identified using (A) *E. voratum* RCC1521 Iso-Seq, (B) *E. voratum* RCC1521 RNA-Seq, (C) *D. trenchii* CCMP2556 RNA-Seq, and (D) *S. microadriaticum* CCMP2457 RNA-Seq datasets, (E) locations of RNA edits, (F) frequency of RNA edits in *D. trenchii* CCMP2556 and in *E. voratum* RCC1521 along relative positions of genes, and (G) distances relative to the next edit within a gene.

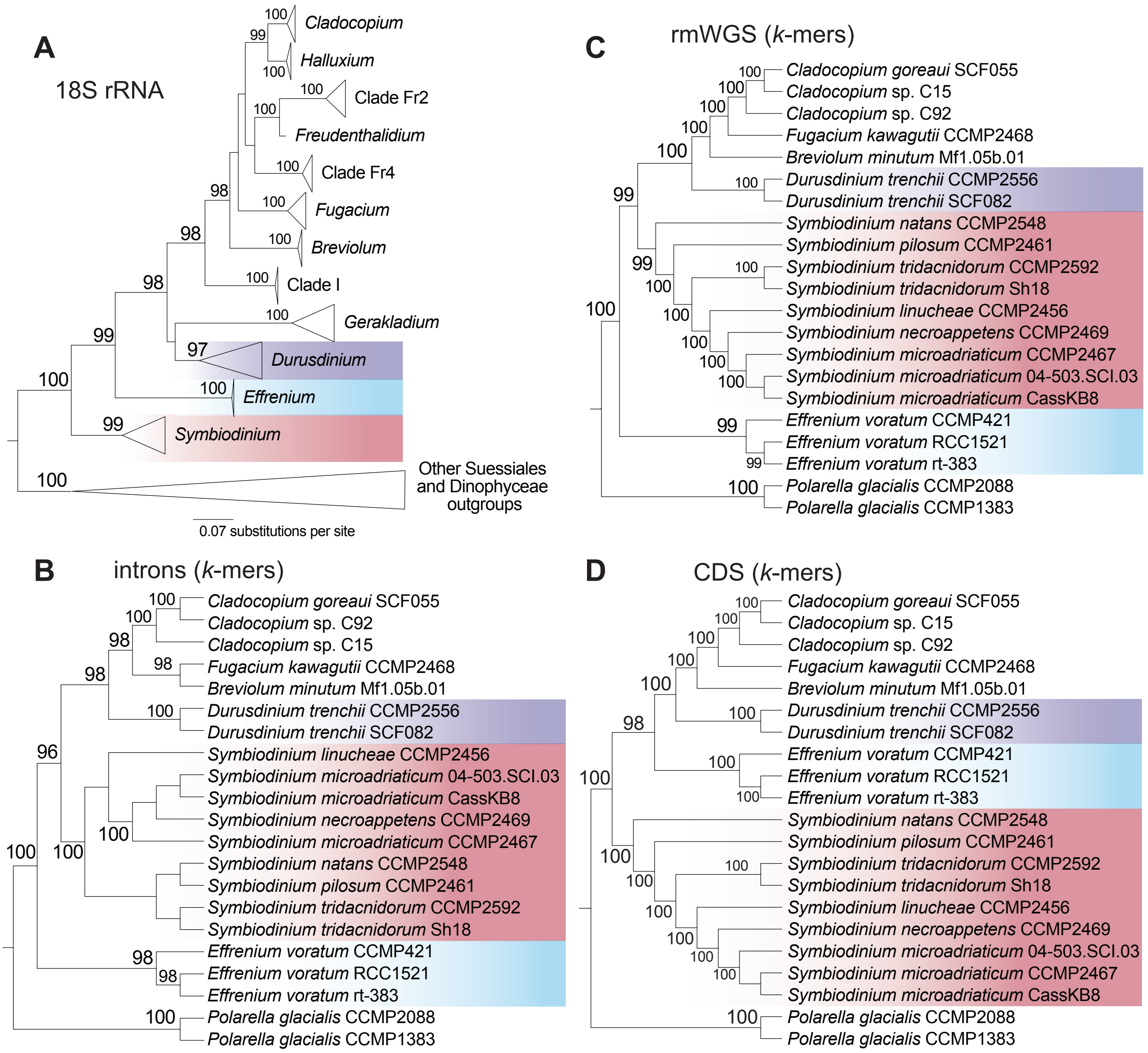

Supplementary Fig. 7. Phylogenetic relationship of *E. voratum* within Symbiodiniaceae and other taxa. (A) Maximum likelihood species tree of dinoflagellates inferred based on alignment of 18S rRNA genes from 13 representative taxa, showing node support derived from ultrafast bootstrap of 2,000 samples; unit of branch length is number of substitutions per site. Tree topologies inferred from *k*-mers in 21 Suessiales genomes using our alignment-free approach are shown for (B) intronic regions ($k = 21$), (C) repeat-masked whole-genome sequences ($k = 23$), and (D) coding sequence regions ($k = 19$). Jackknife support of ≥ 96% based on 100 pseudo-replicates is shown on each node of the AF trees.

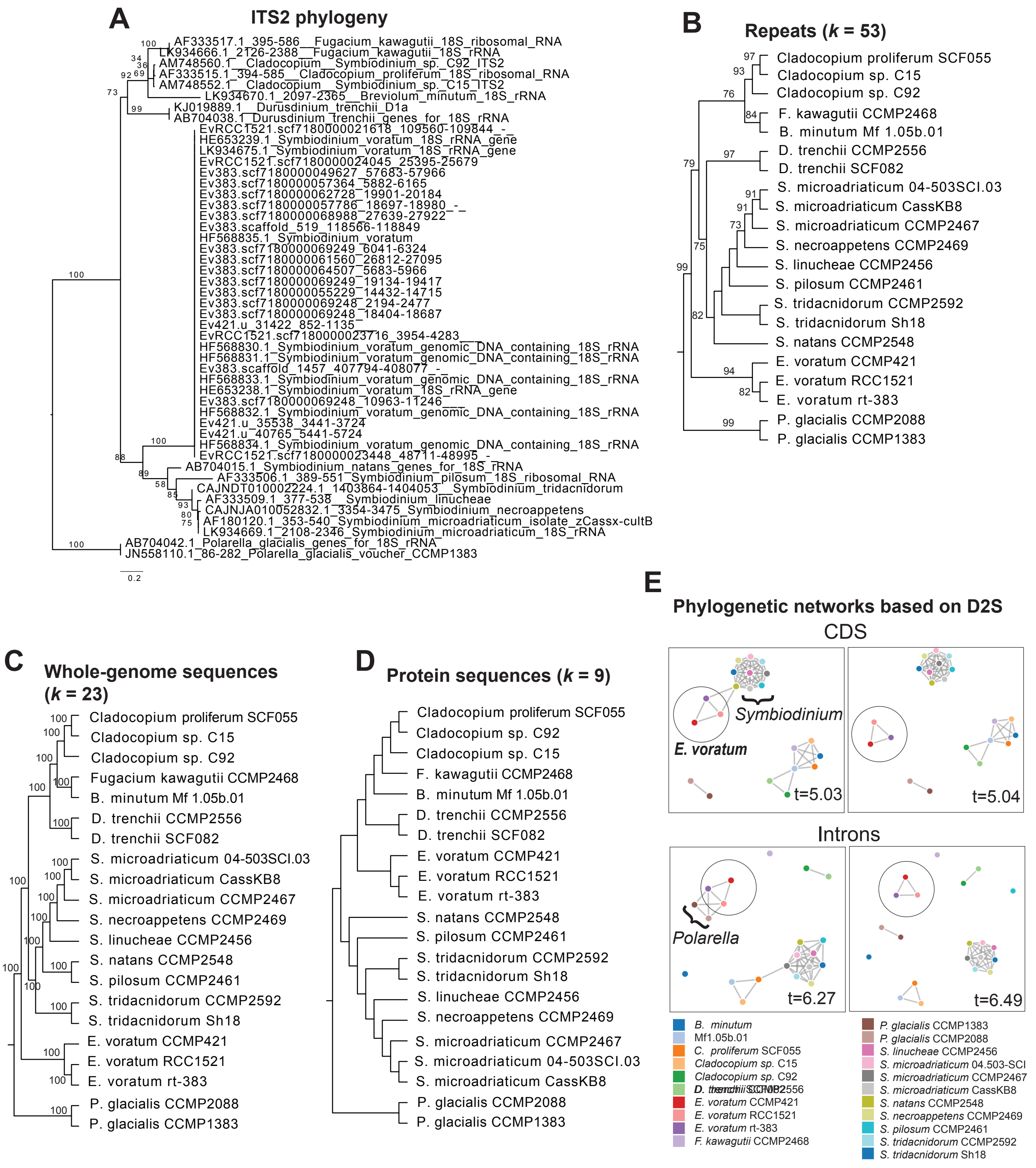

Supplementary Fig. 8. Phylogenetic relationship of Suessiales inferred as trees based on (A) alignment of ITS2 sequences showing ultrafast bootstrap support value from 2,000 samples on each branch (unit in number of substitution per site); as tree topologies based on *k*-mers derived from (B) annotated repeat regions, (C) whole-genome sequences, and (D) protein sequences, showing jackknife support ≥70% from 100 pseudo-replicates where available; and as networks using (E) *k*-mer-derived distances from protein-coding and intron sequences, shown at distinct similarity thresholds *t* relative to split of *E. voratum* from other taxa.

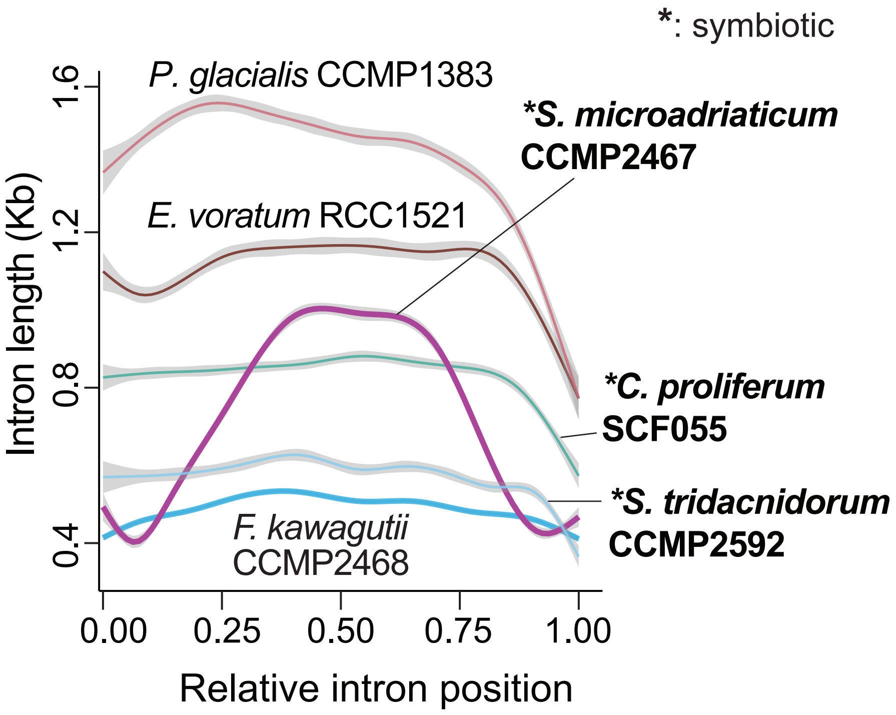

Supplementary Fig. 9. Intron length versus relative intron position in representative Symbiodiniaceae genomes.
