# Supplementary material for: Massive genome reduction predates the divergence of Symbiodiniaceae dinoflagellates
Source: ISME J. 2024 Apr 24;18(1):wrae059. doi: 10.1093/ismejo/wrae059 (PMC11114475; doi:10.1093/ismejo/wrae059)
Supplement: Shah_SupplementaryText_ISMEJ_R3_Final_wrae059 [file shah_supplementarytext_ismej_r3_final_wrae059.pdf]

Supplementary Note

Mobile elements and introner elements

Mobile elements, particularly transposable elements (TEs), can influence genomic architecture and base composition, and have been used to reconstruct the evolutionary history of many species [1]. Although facultative Symbiodiniaceae symbionts (i.e., S1 and S2) are expected to contain a larger proportion of mobile elements in their genomes when compared to free-living (Ev) lineages [2], no significant difference among the groups was observed in the overall abundance (Supplementary Table 12 and Supplementary Fig. 4A) or conservation of TEs (Supplementary Fig. 4B). Although more contiguous genome assemblies generally contain more conserved repeats (Kimura substitution values centred around 3 for *D. trenchii* CCMP2556, *C. proliferum* SCF055 [formerly *C. goreaui* SCF055 [3]], the three *E. voratum* isolates, *S. natans* CCMP2548, and *S. tridacnidorum* CCMP2592) compared to the others (i.e., values centred around 20), we note diverged repeats (Kimura values centred around 25) in the chromosome-scale assembly of *S. microadriaticum* CCMP2467 (Supplementary Fig. 4B). This result suggests a potential technical bias in the recovery of mobile elements. Despite this issue, based on the proportions of distinct types of mobile elements in each group, we found significantly ($P < 0.05$) more long interspersed nuclear elements (LINEs) in S1 (3.9%) compared to Ev (1.8%) and to S2 (1.9%), with the proportion of the outgroup Po at 8% (Fig. 2C). This result suggests that S1 likely retains ancestral LINEs, lending support to the notion of the loss of LINEs after the diversification of the early diverging Symbiodiniaceae genera [4].

Introner elements (IEs) are a type of mobile element consisting of inverted and direct repeat motifs found at 5′- and 3′-end of introns in diverse eukaryotes [5, 6]. Recent research on dinoflagellates revealed that IEs are more abundant in free-living (within 10-12% of genes) than in symbiotic/parasitic species (0.8-6.0% of genes) [7, 8]. Although not as high as in other free-living dinoflagellates, the Ev genomes exhibit more IE-containing genes (5%) than do the S1 (4%) and S2 genomes (3%) (Fig. 2D). IEs have been postulated to be non-autonomous and their mobility is dependent on transposases encoded in dinoflagellate genomes [8]. We recovered transposase protein sequences from most of the Symbiodiniaceae genomes in this study (Supplementary Table 13), suggesting a capacity for IEs to be mobile.

Editing of mRNAs

Editing of mRNAs allows Symbiodiniaceae to increase the variability in protein isoforms [9]. To assess mRNA editing in *E. voratum*, we focused on RCC1521, which has the most contiguous genome assembly, and compared the rates and types of mRNA editing against a representative from S1 (*S. microadriaticum* CCMP2467 [9]) and S2 (*D. trenchii* CCMP2556 [10]). Using a conservative approach to tease apart potential genomic polymorphism and mRNA editing using both transcriptome and genome data [11] (see Supplementary Methods), we identified 45,009 unique mRNA edited sites in *E. voratum*, about 13-fold and 4-fold greater than in *S. microadriaticum* and in *D. trenchii*, respectively (Supplementary Table 17). A larger proportion of genes in *E. voratum* (9,158, 28.5%) contain mRNA edits, compared to *S. microadriaticum* (774, 1.6%) and *D. trenchii* (4,227, 7.6%), although the distribution of substitution types is similar among the three species (Supplementary Figs. 6A–D). Most edits in *D. trenchii* and *E. voratum* (>60%) are located in exons (Supplementary Fig. 6E), whereas in *S. microadriaticum* edits are evenly split between exons and introns, although this may be due to the more-fragmented genome assembly used in Liew et al. [9]. As also observed in *S. microadriaticum* [9], there is a slight bias for edits to occur near 5′ ends of genes (within the first ~10% of gene length, $P < 0.05$; Supplementary Fig. 6F) and the edits tend to be located within 1 Kb of each other (versus the distribution expected at random, $P < 0.05$; Supplementary Fig. 6G). The high level of mRNA editing in Ev is consistent with data from another free-living dinoflagellate, *Pr. cordatum* (42,969 edited sites; 32,067 within 10,169 [12%] genes) [7]. Together with our observation of fewer Ev-specific protein families than those specific to S1 or S2 (Fig. 2), these results suggest a more pronounced role of mRNA editing in generating functional diversity in free-living versus symbiotic dinoflagellate taxa. The relationship and impact of this RNA editing on the encoded proteins remain to be investigated using proteomics.

Impact of symbiogenesis on phylogenetic signal in non-coding regions

Our alignment-based phylogenetic trees that were inferred using multiple protein families (Fig. 1A), the standard molecular marker of 18S rRNA gene (Supplementary Fig. 7A), and the ITS2 region (Supplementary Fig. 8A) were consistent with previously established phylogenies [12-14]. We then analysed the phylogenetic signal of whole-genome sequence data using a *k*-mer-based alignment-free (AF) approach (see Methods), focusing on distinct genome-sequence regions following Lo et al. [15]. The inferred AF phylogenies of introns

(Supplementary Fig. 7B), repetitive regions (Supplementary Fig. 8B), repeat-masked whole-genome sequences (Supplementary Fig. 7C), and entire whole-genome sequences (Supplementary Fig. 8C) placed Ev as the basal group, branching earlier than S1/S2. In comparison, the AF phylogenies for coding regions (i.e., coding sequences [CDS; Supplementary Fig. 7D] and protein sequences [Supplementary Fig. 8D]) were largely congruent with the phylogeny of 18S rRNA gene (Supplementary Fig. 7A), placing S1 as earlier branching than Ev. The branching orders of Ev versus S1/S2 in AF trees were supported by a robust jackknife support of ≥ 96% based on 100 subsampled replicates (Supplementary Figs. 7B–D). This trend is consistent with visualisation of the AF distances as a phylogenomic network (Supplementary Fig. 8E), in which *E. voratum* is more closely related to *P. glacialis* based on intron sequences, and to *Symbiodinium* spp. based on CDS. The incongruence between phylogenies inferred from coding versus non-coding regions with robust support of the distinct clades clearly indicate differential selective pressure acting on these two regions in Symbiodiniaceae genomes, as demonstrated in an earlier study [15].

Supplementary Methods

Extraction of total RNA for RNA-Seq analysis

To increase transcriptome diversity, in addition to the use of QIAGEN RNeasy Plant Mini Kit (Materials and Methods), we extracted more RNA using a second method. In this instance, total RNA was extracted for RNA-Seq (RCC1521 and rt-383) and Iso-Seq (from rt-383) following Acosta-Maspons et al. [16] with slight modifications. Cells were pelleted (300*g*, 5 min), and suspended in lysis buffer (100–500μL; 100mM Tris-HCl, 25mM EDTA, 2% CTAB w/v, 2M NaCl, 0.75 g/L spermidine trihydrochloride, 4% β-mercaptoethanol). This mixture ground using a pre-chilled mortar and pestle to fine powder, swirled with liquid nitrogen, and transferred to a chilled 15mL Falcon tube. Lysis buffer (5mL, 65°C) was added, and an equal volume of chloroform:isoamyl alcohol (24:1 v/v) was mixed in. The mixture was divided into aliquots (2mL) in Eppendorf tubes and centrifuged (10000*g*, 10 min, 4°C). The clear supernatants were subjected to another round of chloroform:isoamyl alcohol (24:1 v/v) extraction. Nucleic acids were precipitated using LiCl (final concentration 2M) overnight at 4°C. The pellet collected via centrifugation (17000*g*, 30min, 4°C) was resuspended in the STE buffer (50 μL; 1M NaCl, 0.5% SDS w/v, 10mM Tris-HCl pH 8, 1mM EDTA, 65°C); the samples were not set on ice to avoid undesirable precipitates. Further chloroform:isoamyl alcohol (24:1 v/v) extraction was performed (1000*g*, 10min, 4°C), RNA was precipitated using an equal volume of isopropanol (10min, RT) before centrifugation (17000*g*, 10min, 4°C). The pellet was washed with 80% ethanol (500μL), dislodged by pulse vortexing (2s), and the tube centrifuged (17000*g*, 5min, 4°C); this step was repeated before the final pellet was air-dried (10min) in a fume hood, resuspended nuclease-free water (25μL) and stored at –80°C.

Generation of genome data

For short-read sequencing, the libraries for RCC1521 and rt-383 were prepared using the Illumina TruSeq Nano kit with 350 bp targeted inserts following standard protocol, and sequenced on the NovaSeq 6000 platform at Australian Genome Research Facility (Melbourne, Australia). For CCMP421, the genomic DNA library was prepared using the Chromium Genome Reagent Kit v2 Chemistry following the manufacturer's protocol (Step 2 GEM generation and barcoding onwards) and sequenced on the NovaSeq 6000 platform at Florida International University.

For Nanopore sequencing of RCC1521 and rt-383, the ligation kit SQK LSK-109 was used following standard protocol. Each library was sequenced on a MinION flow cell, and rt-383 gDNA was further sequenced using a PromethION flow cell at the University of Queensland Genome Innovation Hub (Brisbane, Australia). The sequence reads were base-called using guppy v4.0.11 of MinKNOW v20.06.18 (minimum read quality filter = 7).

For PacBio sequencing, the gDNA of RCC1521 (unsheared, for continuous long-read [CLR] library) and rt-383 (sheared in 15–20Kb fragments with Pippin Prep [Sage Science] for HiFi library) were used for library preparation using the SMRTbell Express Template Prep Kit 2.0 following the manufacturer's protocol. The RCC1521 and the rt-383 libraries were sequenced on the PacBio Sequel II platform, respectively at the University of Washington PacBio Sequencing Services (Seattle, WA, USA) and at the University of Queensland Sequencing Facility (Brisbane, Australia). CLR reads were acquired using the PacBio BAM2fastx toolkit. HiFi consensus reads were obtained using the CCS module of SMRT Link pipeline v8.0.

Generation of transcriptome data

Transcriptome data were generated for RCC1521 and rt-383. Illumina RNA-Seq libraries were generated using polyA-selection with the Dynabeads mRNA purification Kit (Thermo Fisher) and the Illumina Stranded mRNA Prep following standard protocols. Sequencing was performed on the Illumina NovaSeq 6000 platform at the Australian Genome Research Facility (Melbourne, Australia). PacBio Iso-Seq libraries were prepared using the NEBNext® Single Cell/Low Input cDNA Synthesis and Amplification Module (New England BioLabs) and the SMRTbell Express Template Prep Kit 2.0 following standard protocol and sequenced on the PacBio Sequel II at the University of Queensland Sequencing Facility (Brisbane, Australia).

Estimation of genome size from sequencing data

Illumina short reads were used for estimating genome size based on *k*-mers. The reads from each genome dataset were processed to remove potential adapters (for 10X linked-reads of CCMP421, specifically the first 23 bases) and polyG tails using fastp v0.20.0 [17]. Jellyfish v2.3.0 [18] was used to obtain *k*-mers, independently for each odd *k* value between 17 and 31, inclusive. Genome sizes were estimated using the *k*-mer distributions, as the sum of observed *k*-mers divided by the *k*-mer coverage corresponding to peak of the distribution

(Supplementary Table 9). Ploidy of each genome dataset was assessed using GenomeScope2 [19] based on 21-mer distribution, with better fit observed for haploid model (p=1) in all three *E. voratum* genomes. Genome size estimations of other dinoflagellates (Supplementary Table 8) were obtained from earlier studies [20-23].

Identification of organellar genome sequences

To identify mitochondrial genome sequences, we followed Stephens et al. [24] by adopting BLASTn v2.10.0 [25] search ($E \leq 10^{-10}$) against the assembled genomes, using known mitochondrial protein-coding sequences (GenBank accessions LC002801.1 and LC002802.1) as queries. We used BEDtools [26] *merge* to merge overlapping BLAST hits (*-s -o collapse -c 1,2,3,4,5,6*), and BEDtools *intersect* (*-wa -wb*) to check for overlaps of gene annotations. The resulted genome scaffolds that did not have other predicted genes were considered putative mitochondrial genome sequences.

To identify plastid genome sequences, we performed an independent short-read only genome assembly for each isolate using CLC Genomics Workbench v21.0.4 (Supplementary Table 5). We used BLASTn search ($E \leq 10^{-10}$) using protein-coding sequences of plastid-encoded genes for *Cladocopium* sp. C3 (GenBank accessions HG515015.1-HG515028.1) as query, and annotated protein-coding genes using Artemis [27] (translation table 11). The conserved core region of these sequences was used as query in BLASTn searches among other short-read-assembled genome sequences to identified putative empty minicircles. Evidence of circularisation was accessed using nucmer (*--mum -l 0*) and mummerplot (*--layout --png*) in MUMmer v4.0.0beta2 [28].

Ab initio **prediction of protein-coding genes**

To predict protein-coding genes, we used a workflow customised for dinoflagellates following Chen et al. [29], incorporating protein and transcriptome evidence and multiple predictors (https://github.com/TimothyStephens/Dinoflagellate_Annotation_Workflow).

For each genome assembly, *de novo* repeat families were first predicted using RepeatModeler v2.0.1 [30]. These repeats were added to the Dfam database (dfam.org; downloaded June 2019) to guide RepeatMasker v4.1.0 (https://www.repeatmasker.org/) in masking repetitive genomic regions. GeneMark-ES v4.65 [31] was used for *ab initio* gene prediction on the masked genome assembly. Protein-based gene prediction was performed on the unmasked

genome assembly, using the *protein2genome* of MAKER v2.31.10 [32] that was modified to recognise dinoflagellate alternative splice sites, and a comprehensive protein sequence database combining SwissProt (release March 2020) and the Suessiales sequences, hereby "Suessiales_pep" (Supplementary Table 6), following Chen et al. [29].

For transcript-based gene prediction, Iso-Seq transcripts where available (i.e., for RCC1521 and rt-383) were mapped on the corresponding genome assembly using minimap2 v2.18 for which the code was modified to recognise dinoflagellate alternative splice sites, with *--secondary=no -ax splice:hq -uf --splice-flank=no.* The assembled transcripts from RNA-Seq (both *de novo* and genome-guided) for each isolate were mapped to the corresponding genome assembly using BLAT [33]; for CCMP421, *de novo* assembled transcripts from RCC1521 and rt-383, plus the assembly of these reads guided by the CCMP421 genome, were used in this step. The resulting GFF3 files were input into PASA v2.4.1 [34] modified to recognise dinoflagellate alternative splice sites, with options *--IMPORT_CUSTOM_ALIGNMENTS_GFF3 --transcribed_is_aligned_orient -C -R --MAX_INTRON_LENGTH 70000.*

The PASA-predicted genes were filtered in the following steps: they were searched against the database combining RefSeq (release 98) and Suessiales_pep (above) using BLASTp v2.2.26 ($E < 10^{-20}$, both query and subject coverages > 80%) [25], putative transposon sequences were removed via running HHBLITS v3.3.0 [35] and TransposonPSI v1.0.0 (https://sourceforge.net/projects/transposonpsi/) against the UniRef30_2020_03 database (https://uniclust.mmseqs.com/) [36], redundant sequences were removed using CD-HIT v4.8.1 (*-c 0.75 -n 5*) [37], and the script *Prepare_golden_genes_for_predictors.pl* in JAMg pipeline (https://github.com/genomecuration/JAMg) was used to produce a highly curated set of "golden genes". These golden genes were used to guide the *ab initio* gene predictions using SNAP [38] and AUGUSTUS v3.4.0 [39] on the repeat-masked genome assembly. Gene models from the five tools (GeneMark-ES, MAKER, PASA, SNAP, AUGUSTUS) were integrated using EVidenceModeler v1.1.1 [40], following Chen et al. [41]. Finally, the resulting gene models were refined to correct exon boundaries, identify untranslated regions, and incorporate alternative splice-forms using the *Load_Current_Gene_Annotations.dbi* and *Launch_PASA_pipeline.pl* from the PASA pipeline [42, 43] iteratively for three rounds to yield the final gene models.

We assessed the completeness of the predicted protein sequences using BUSCO v5.1.2 [44] against the alveolata_odb10 (*protein* mode). Genes with transcript support were assessed BLASTn ($E < 10^{-5}$, percent identity ≥ 90%, subject cover ≥ 50%).

Consistent functional annotation of predicted genes

To ensure comparability of gene functions among different genomes, we annotated functions of all proteins predicted from the 21 Suessiales genomes (Supplementary Table 7) using a consistent approach. Functions of protein sequences were annotated based on BLASTp searches (e-value < 10^{-5}, query/subject cover ≥ 50 %) against SwissProt (2022_01). Those that had no hits were searched against TrEMBL (2022_01); the function of the top protein hit was assumed to the putative function of the query protein. Gene Ontology (GO) terms associated with each UniProt identifier were acquired using the UniProtKB ID mapping tool (https://www.uniprot.org/id-mapping; December 2022).

Enrichment of Gene Ontology terms

For the analysis of gene family evolution of Suessiales taxa (Materials and Methods), Gene Ontology (GO) terms enrichment was performed for six comparisons: (a) shared genes in Ev+Po (test set) versus all genes in Ev+Po (background), (b) shared genes in S1+S2 versus all genes in S1 and S2, (c) shared genes in S1+S2+Po versus all genes in S1, S2, and Po, (d) shared genes in S1+S2+Ev+Po versus all genes in the 21 taxa, (e) exclusive genes to S1 versus all S1 genes, and (f) genes exclusive to Ev versus all Ev genes.

Inference of species tree

To reconstruct a species tree of dinoflagellates based on strictly orthologous protein sequences, we incorporated 1,603,073 predicted protein sequences from 33 dinoflagellate taxa, comprising 21 Suessiales taxa (including the three *E. voratum* isolates) and 12 other taxa external to Suessiales (Supplementary Table 8). These sequences were clustered into homologous sets using OrthoFinder v2.5.4 [45], from which a species tree was estimated from strictly orthologous sets.

Alignment-free phylogenetic inference and core *k*-mers

Alignment-free (AF) approach was used to infer phylogenetic relationships from (a) whole-genome sequences (WGS) and from distinct genomic regions of (b) repeat-masked WGS, (c)

coding sequences (CDS), (d) introns, (e) annotated repeats, and (f) predicted protein sequences. Each of these distinct regions were extracted from assembled genome sequences using *gff3_file_to_feature_files.pl* implemented in PASA [34]. We followed Lo et al. [15] to identify optimal *k*-mer length (*k*) for each of these datasets. Briefly, for each dataset, *k*-mers at varied length *k* were enumerated using Jellyfish v2.3.0 [18]; for all datasets, odd-numbered *k* between 13 and 27 were used, except for the repeats dataset (odd-numbered *k* values between 13 and 51 were used) and protein sequences (odd-numbered *k* values between 3 and 9 were used). For each dataset except the protein sequences, the optimal *k* was determined based on the cumulative proportion of unique *k*-mers and the cumulative proportion of distinct *k*-mers, at the point when distributions of both proportions reached a plateau (Supplementary Fig. 1); *k* value determined this way was found to yield the greatest distinguishing power for phylogenetic analysis [46]. For protein sequences, we followed Lo et al. [15] to infer AF phylogenies (below) from each *k* and chose the *k* with a topology that best matched the alignment-based tree of the 18S rRNA genes [15]. The optimal *k* was identified as 23 for WGS and repeat-masked WGS, 19 for CDS, 21 for introns, 51 for annotated repeats, and 9 for protein sequences. Jellyfish v2.3.0 was used to extract *k*-mers at the corresponding optimal *k* length for each dataset; option *-C* was used to enforce strand-specific directionality for the intron and CDS datasets.

To infer AF phylogenies based on *k*-mers (i.e., using the optimal *k* for each corresponding dataset identified above), we derived pairwise distance based on D_2^S statistic [47] following Chan et al. [48], using *d2ssect* (https://github.com/bakeronit/d2ssect). These pairwise distances were then used to infer a phylogenetic tree using *neighbor* implemented in PHYLIP v3.698 (https://evolution.gs.washington.edu/phylip.html). For each tree, we assessed node support based on jackknife analysis of 100 "pseudo-replicates" following Bernard et al. [49]. In each pseudo-replicate, 40% of the data, in iteratively subsampled 100-bp regions at random, were deleted using the Python script *jackknife.py* from which an AF tree was inferred; the R script *Jackknife.r* was then used to calculate jackknife support value in percentage, among the pseudo-replicate trees, for each node in the original AF tree. These scripts are available at https://github.com/chanlab-genomics/alignment-free-tools.

To identify core *k*-mers [15, 50] that are shared by all 21 Suessiales genomes used in this study, we used the optimal *k* = 23 for the WGS dataset. Using the extracted 23-mers from the entire WGS dataset as input, core 23-mers were identified using the bash command *comm* (-

12). BEDtools [26] *intersect* was used to identify overlaps between the core *k*-mers and annotated genomic features.

Analysis of mRNA editing

For the analysis of mRNA editing, we focused on *E. voratum* RCC1521, and the representative genomes for S1 (*S. microadriaticum* CCMP2467) and S2 (*D. trenchii* CCMP2556), for which high-quality genome and transcriptome data are available. Editing of mRNAs was identified using JACUSA2 [11], based on observed nucleotide variants in the mapping of transcripts onto the genome, relative to the mapping of genome sequence reads onto the genome. For each genome, the gDNA reads were mapped on the genome assembly using BWA-mem v0.7.17-r1198 [51] at default setting. RNA-Seq reads were then mapped using HISAT2 v2.2.0 [52] *(--rna-strandness RF*) against the genome assembly, with a HGFM HISAT2 index (*hisat2-build --exon --ss*) informed by the annotated splice sites. To generate this index, the gene annotation file in GFF3 was converted to the GTF format using Gffread [53], and splice site and exon locations extracted with the *hisat2_extract_splice_sites.py* and *hisat2_extract_exons.py* scripts. Iso-Seq reads, where available, were mapped using minimap2 v2.18 [54] for which the code was modified to recognise alternative splice sites of dinoflagellates, with options *--splice-flank=no –secondary=no -ax splice:hq -uf --junc-bed*.

Duplicate mappings were removed from the gDNA BAM files using Picard *MarkDuplicates* (*ASSUME_SORTED=true REMOVE_DUPLICATES=true CREATE_INDEX=TRUE VALIDATION_STRINGENCY=LENIENT*). The MD field documenting mismatched and deleted bases was added to the gDNA BAM files using samtools *calmd* (*-b*), as required as input for JACUSA2. JACUSA2 analysis was performed on the gDNA, RNA-Seq, and Iso-Seq BAM files using option *-a D,Y,H* to remove false positives caused by read starts/ends, indels, splice sites, and homopolymers. Due to the different strand directionality, *-P2 RF-FIRSTSTRAND* was specified for the runs incorporating RNA-Seq data, and *-P2 FR-SECONDSTRAND* for the runs using Iso-Seq data. The overlap between RNA edited sites and gene models (and isoforms) was identified using BEDtools [26] *intersect* at *-s -wo*.

We followed Liew et al. [9] to assess 5′ bias in the location of RNA editing and the propensity for edits to occur together. We calculated the frequency of the locations of each edit with respect to the features they were in (exon, intron, gene), normalised by the length of

each feature. For each edit, its distance (in bp) to the closest upstream edit, and that to the closest downstream edit where all within the same gene, were determined. The average of these two values was used as the per-edit observed distance to neighbouring edits.

Analysis of introner elements

To identify introner elements (IEs), we used Pattern Locator [55] following Farhat et al. [8]: inverted repeats of 8-20 nucleotides within 30 bases of the 5′ and 3′ ends of each intron, flanked by direct repeats of 3–5 nucleotides. We used Seqkit to obtain the first and last 30 bases at each end of introns, then Pattern Locator to identify the IEs.

Analysis of lineage-specific protein sets

To assess if our observation of lineage-specific protein sets was caused by the unbalanced representation of protein datasets among the three groups (i.e., 9 taxa in S1, 7 in S2, and 3 in Ev), we examined the number of lineage-specific protein sets across 100 independent tests. For each test, among the 811,661 protein sequences from the 21 Suessiales datasets, three datasets from S1 and three from S2 were randomly sampled and included, to achieve the balanced representation of 3 datasets per group; the outgroup Po remains as 2 since we only have data from two isolates of *Polarella glacialis*. The protein sequences from these 11-taxon datasets were then clustered into homologous sets using OrthoFinder v2.5.4 [45] at default setting. Of the 100 tests, majority yielded more S1-specific sets than Ev-specific sets (64 cases), and more S2-specific sets than Ev-specific sets (69), whereas in 43 cases, both S1-specific and S2-specific sets are greater than Ev-specific sets (Supplementary Table 15). These numbers, while do not meet the usual expectation of a statistical significance (e.g. >90%), provide an insight into how sampling bias of Symbiodiniaceae genomes could affect our results. On the other hand, we observed greater number of protein sets shared by Po with S1 and S2 (i.e. S1+S2+Po) than with Ev (i.e. Ev+Po) in 99 of the 100 cases, indicating statistical robustness of this trend based on the datasets analysed. Based on these results, we caution that the number of lineage-specific protein sets need to be interpreted in the appropriate context, and not taken at face value. Protein sets identified this way, based on sequence similarity using OrthoFinder, represent a proxy for studying evolution of protein or gene families. Given that Ev consists only sequences from one species, whereas those in S1 and S2 comprise datasets of different species and genera, greater divergence of protein sequences in these groups compared to Ev is reasonable, as reflected in our results.

Supplementary references

1. Cai L, Arnold BJ, Xi Z, Khost DE, Patel N, Hartmann CB, et al.; Deeply altered genome architecture in the endoparasitic flowering plant *Sapria himalayana* Griff. (Rafflesiaceae). *Curr Biol* 2021;**31**(5):1002-1011.e9. doi: 10.1016/j.cub.2020.12.045.

2. González-Pech RA, Bhattacharya D, Ragan MA, Chan CX; Genome evolution of coral reef symbionts as intracellular residents. *Trends Ecol Evol* 2019;**34**(9):799-806. doi: 10.1016/j.tree.2019.04.010.

3. Butler CC, Turnham KE, Lewis AM, Nitschke MR, Warner ME, Kemp DW, et al.; Formal recognition of host-generalist species of dinoflagellate (*Cladocopium*, Symbiodiniaceae) mutualistic with Indo-Pacific reef corals. *J Phycol* 2023;**59**(4):698-711. doi: 10.1111/jpy.13340.

4. González-Pech RA, Stephens TG, Chen Y, Mohamed AR, Cheng Y, Shah S, et al.; Comparison of 15 dinoflagellate genomes reveals extensive sequence and structural divergence in family Symbiodiniaceae and genus *Symbiodinium*. *BMC Biol* 2021;**19**(1):73. doi: 10.1186/s12915-021-00994-6.

5. Worden AZ, Lee J-H, Mock T, Rouzé P, Simmons MP, Aerts AL, et al.; Green evolution and dynamic adaptations revealed by genomes of the marine picoeukaryotes *Micromonas*. *Science* 2009;**324**(5924):268-272. doi: 10.1126/science.1167222.

6. Gozashti L, Roy SW, Thornlow B, Kramer A, Ares M, Corbett-Detig R; Transposable elements drive intron gain in diverse eukaryotes. *Proc Natl Acad Sci U S A* 2022;**119**(48):e2209766119. doi: 10.1073/pnas.2209766119.

7. Dougan KE, Deng Z-L, Wöhlbrand L, Reuse C, Bunk B, Chen Y, et al.; Multi-omics analysis reveals the molecular response to heat stress in a “red tide” dinoflagellate. *Genome Biol* 2023;**24**:265. doi: 10.1186/s13059-023-03107-4.

8. Farhat S, Le P, Kayal E, Noel B, Bigeard E, Corre E, et al.; Rapid protein evolution, organellar reductions, and invasive intronic elements in the marine aerobic parasite dinoflagellate *Amoebophrya* spp. *BMC Biol* 2021;**19**(1):1. doi: 10.1186/s12915-020-00927-9.

9. Liew YJ, Li Y, Baumgarten S, Voolstra CR, Aranda M; Condition-specific RNA editing in the coral symbiont *Symbiodinium microadriaticum*. *PLoS Genet* 2017;**13**(2):e1006619. doi: 10.1371/journal.pgen.1006619.

10. Dougan KE, Bellantuono AJ, Kahlke T, Abbriano RM, Chen Y, Shah S, et al.; Whole-genome duplication in an algal symbiont serendipitously confers thermal tolerance to corals. *bioRxiv* 2022:2022.04.10.487810. doi: 10.1101/2022.04.10.487810.

11. Piechotta M, Naarmann-de Vries IS, Wang Q, Altmuller J, Dieterich C; RNA modification mapping with JACUSA2. *Genome Biol* 2022;**23**(1):115. doi: 10.1186/s13059-022-02676-0.

12. LaJeunesse TC, Parkinson JE, Gabrielson PW, Jeong HJ, Reimer JD, Voolstra CR, et al.; Systematic revision of Symbiodiniaceae highlights the antiquity and diversity of coral endosymbionts. *Curr Biol* 2018;**28**(16):2570-2580.e6. doi: 10.1016/j.cub.2018.07.008.

13. Price DC, Bhattacharya D; Robust Dinoflagellata phylogeny inferred from public transcriptome databases. *J Phycol* 2017;**53**(3):725-729. doi: 10.1111/jpy.12529.

14. Stephens TG, Ragan MA, Bhattacharya D, Chan CX; Core genes in diverse dinoflagellate lineages include a wealth of conserved dark genes with unknown functions. *Sci Rep* 2018;**8**(1):17175. doi: 10.1038/s41598-018-35620-z.

15. Lo R, Dougan KE, Chen Y, Shah S, Bhattacharya D, Chan CX; Alignment-free analysis of whole-genome sequences from Symbiodiniaceae reveals different phylogenetic signals in distinct regions. *Front Plant Sci* 2022;**13**:815714. doi: 10.3389/fpls.2022.815714.

16. Acosta-Maspons A, González-Lemes I, Covarrubias AA; Improved protocol for isolation of high-quality total RNA from different organs of *Phaseolus vulgaris* L. *BioTechniques* 2019;**66**(2):96-98. doi: 10.2144/btn-2018-0129.

17. Chen S, Zhou Y, Chen Y, Gu J; fastp: an ultra-fast all-in-one FASTQ preprocessor. *Bioinformatics* 2018;**34**(17):i884-i890. doi: 10.1093/bioinformatics/bty560.

18. Marçais G, Kingsford C; A fast, lock-free approach for efficient parallel counting of occurrences of *k*-mers. *Bioinformatics* 2011;**27**(6):764-770. doi: 10.1093/bioinformatics/btr011.

19. Ranallo-Benavidez TR, Jaron KS, Schatz MC; GenomeScope 2.0 and Smudgeplot for reference-free profiling of polyploid genomes. *Nat Commun* 2020;**11**(1):1432. doi: 10.1038/s41467-020-14998-3.

20. Rizzo PJ, Jones M, Ray SM; Isolation and properties of isolated nuclei from the Florida red tide dinoflagellate *Gymnodinium breve* (Davis). *J Protozool* 1982;**29**(2):217-222. doi: 10.1111/j.1550-7408.1982.tb04014.x.

21. Hou Y, Lin S; Distinct gene number-genome size relationships for eukaryotes and non-eukaryotes: gene content estimation for dinoflagellate genomes. *PLoS ONE* 2009;**4**(9):e6978. doi: 10.1371/journal.pone.0006978.

22. Sano J, Kato KH; Localization and copy number of the protein-coding genes actin, α-Tubulin, and HSP90 in the nucleus of a primitive dinoflagellate, *Oxyrrhis marina*. *Zool Sci* 2009;**26**(11):745-753. doi: 10.2108/zsj.26.745.

23. Kohli GS, John U, Figueroa RI, Rhodes LL, Harwood DT, Groth M, et al.; Polyketide synthesis genes associated with toxin production in two species of *Gambierdiscus* (Dinophyceae). *BMC Genomics* 2015;**16**(1):410. doi: 10.1186/s12864-015-1625-y.

24. Stephens TG, González-Pech RA, Cheng Y, Mohamed AR, Burt DW, Bhattacharya D, et al.; Genomes of the dinoflagellate *Polarella glacialis* encode tandemly repeated single-exon genes with adaptive functions. *BMC Biol* 2020;**18**(1):56. doi: 10.1186/s12915-020-00782-8.

25. Camacho C, Coulouris G, Avagyan V, Ma N, Papadopoulos J, Bealer K, et al.; BLAST+: architecture and applications. *BMC Bioinformatics* 2009;**10**(1):421. doi: 10.1186/1471-2105-10-421.

26. Quinlan AR, Hall IM; BEDTools: a flexible suite of utilities for comparing genomic features. *Bioinformatics* 2010;**26**(6):841-2. doi: 10.1093/bioinformatics/btq033.

27. Carver T, Harris SR, Berriman M, Parkhill J, McQuillan JA; Artemis: an integrated platform for visualization and analysis of high-throughput sequence-based experimental data. *Bioinformatics* 2011;**28**(4):464-469. doi: 10.1093/bioinformatics/btr703.

28. Marçais G, Delcher AL, Phillippy AM, Coston R, Salzberg SL, Zimin A; MUMmer4: A fast and versatile genome alignment system. *PLoS Comput Biol* 2018;**14**(1):e1005944. doi: 10.1371/journal.pcbi.1005944.

29. Chen Y, Shah S, Dougan KE, van Oppen MJH, Bhattacharya D, Chan CX; Improved *Cladocopium goreaui* genome assembly reveals features of a facultative coral symbiont and the complex evolutionary history of dinoflagellate genes. *Microorganisms* 2022;**10**(8):1662. doi: 10.3390/microorganisms10081662

30. Flynn JM, Hubley R, Goubert C, Rosen J, Clark AG, Feschotte C, et al.; RepeatModeler2 for automated genomic discovery of transposable element families. *Proc Natl Acad Sci U S A* 2020;**117**(17):9451. doi: 10.1073/pnas.1921046117.

31. Lomsadze A, Ter-Hovhannisyan V, Chernoff YO, Borodovsky M; Gene identification in novel eukaryotic genomes by self-training algorithm. *Nucleic Acids Res* 2005;**33**(20):6494-6506. doi: 10.1093/nar/gki937.

32. Cantarel BL, Korf I, Robb SMC, Parra G, Ross E, Moore B, et al.; MAKER: an easy-to-use annotation pipeline designed for emerging model organism genomes. *Genome Res* 2008;**18**(1):188-196. doi: 10.1101/gr.6743907.

33. Kent WJ; BLAT--the BLAST-like alignment tool. *Genome Res* 2002;**12**(4):656-64. doi: 10.1101/gr.229202.

34. Haas BJ, Delcher AL, Mount SM, Wortman JR, Smith RK, Jr., Hannick LI, et al.; Improving the *Arabidopsis* genome annotation using maximal transcript alignment assemblies. *Nucleic Acids Res* 2003;**31**(19):5654-5666. doi: 10.1093/nar/gkg770.

35. Remmert M, Biegert A, Hauser A, Söding J; HHblits: lightning-fast iterative protein sequence searching by HMM-HMM alignment. *Nat Methods* 2012;**9**(2):173-175. doi: 10.1038/nmeth.1818.

36. Mirdita M, von den Driesch L, Galiez C, Martin MJ, Söding J, Steinegger M; Uniclust databases of clustered and deeply annotated protein sequences and alignments. *Nucleic Acids Res* 2017;**45**(D1):D170-D176. doi: 10.1093/nar/gkw1081.

37. Li W, Godzik A; CD-HIT: a fast program for clustering and comparing large sets of protein or nucleotide sequences. *Bioinformatics* 2006;**22**(13):1658-1659. doi: 10.1093/bioinformatics/btl158.

38. Korf I; Gene finding in novel genomes. *BMC Bioinformatics* 2004;**5**(1):59. doi: 10.1186/1471-2105-5-59.

39. Stanke M, Keller O, Gunduz I, Hayes A, Waack S, Morgenstern B; AUGUSTUS: ab initio prediction of alternative transcripts. *Nucleic Acids Res* 2006;**34**(suppl_2):W435-W439. doi: 10.1093/nar/gkl200.

40. Haas BJ, Salzberg SL, Zhu W, Pertea M, Allen JE, Orvis J, et al.; Automated eukaryotic gene structure annotation using EVidenceModeler and the Program to Assemble Spliced Alignments. *Genome Biol* 2008;**9**(1):R7. doi: 10.1186/gb-2008-9-1-r7.

41. Chen Y, Gonzalez-Pech RA, Stephens TG, Bhattacharya D, Chan CX; Evidence that inconsistent gene prediction can mislead analysis of dinoflagellate genomes. *J Phycol* 2020;**56**(1):6-10. doi: 10.1111/jpy.12947.

42. Campbell MA, Haas BJ, Hamilton JP, Mount SM, Buell CR; Comprehensive analysis of alternative splicing in rice and comparative analyses with *Arabidopsis*. *BMC Genomics* 2006;**7**(1):327. doi: 10.1186/1471-2164-7-327.

43. Haas BJ; Analysis of alternative splicing in plants with bioinformatics tools. In: Reddy ASN, Golovkin Ms (eds). *Nuclear pre-mRNA Processing in Plants*. Berlin, Heidelberg: Springer Berlin Heidelberg, 2008, 17-37.

44. Simão FA, Waterhouse RM, Ioannidis P, Kriventseva EV, Zdobnov EM; BUSCO: assessing genome assembly and annotation completeness with single-copy orthologs. *Bioinformatics* 2015;**31**(19):3210-3212. doi: 10.1093/bioinformatics/btv351.

45. Emms DM, Kelly S; OrthoFinder: phylogenetic orthology inference for comparative genomics. *Genome Biol* 2019;**20**(1):238. doi: 10.1186/s13059-019-1832-y.

46. Greenfield P, Roehm U; Answering biological questions by querying *k*-mer databases. *Concurr Comput Pract Exper* 2013;**25**(4):497-509. doi: 10.1002/cpe.2938.

47. Reinert G, Chew D, Sun F, Waterman MS; Alignment-free sequence comparison (I): statistics and power. *J Comput Biol* 2009;**16**(12):1615-1634. doi: 10.1089/cmb.2009.0198.

48. Chan CX, Bernard G, Poirion O, Hogan JM, Ragan MA; Inferring phylogenies of evolving sequences without multiple sequence alignment. *Sci Rep* 2014;**4**(1):6504. doi: 10.1038/srep06504.

49. Bernard G, Chan CX, Ragan MA; Alignment-free microbial phylogenomics under scenarios of sequence divergence, genome rearrangement and lateral genetic transfer. *Sci Rep* 2016;**6**(1):28970. doi: 10.1038/srep28970.

50. Bernard G, Greenfiled P, Ragan MA, Chan CX; *k*-mer similarity, networks of microbial genomes, and taxonomic rank. *mSystems* 2018;**3**(6):e00257-18. doi: 10.1128/mSystems.00257-18.

51. Li H, Durbin R; Fast and accurate short read alignment with Burrows-Wheeler transform. *Bioinformatics* 2009;**25**(14):1754-60. doi: 10.1093/bioinformatics/btp324.

52. Kim D, Paggi JM, Park C, Bennett C, Salzberg SL; Graph-based genome alignment and genotyping with HISAT2 and HISAT-genotype. *Nat Biotechnol* 2019;**37**(8):907-915. doi: 10.1038/s41587-019-0201-4.

53. Pertea G, Pertea M; GFF Utilities: GffRead and GffCompare [version 2; peer review: 3 approved]. *F1000Res* 2020;**9**:304. doi: 10.12688/f1000research.23297.2.

54. Li H; Minimap2: pairwise alignment for nucleotide sequences. *Bioinformatics* 2018;**34**(18):3094-3100. doi: 10.1093/bioinformatics/bty191.

55. Mrázek J, Xie S; Pattern locator: a new tool for finding local sequence patterns in genomic DNA sequences. *Bioinformatics* 2006;**22**(24):3099-3100. doi: 10.1093/bioinformatics/btl551.
